# Hepatocyte nuclear factor 1 alpha influences pancreatic cancer growth and metastasis

**DOI:** 10.1038/s41598-020-77287-5

**Published:** 2020-11-19

**Authors:** Ramadevi Subramani, Joshua Medel, Kristina Flores, Courtney Perry, Adriana Galvez, Mayra Sandoval, Servando Rivera, Diego A. Pedroza, Elizabeth Penner, Mahika Chitti, Rajkumar Lakshmanaswamy

**Affiliations:** 1 Center of Emphasis in Cancer Research, Department of Molecular and Translational Medicine, Texas Tech University Health Sciences Center El Paso, Paul L. Foster School of Medicine, El Paso, TX 79905 USA; 2 Graduate School of Biomedical Sciences, Texas Tech University Health Sciences Center, El Paso, TX 79905 USA; 3 Division of Cancer Control and Population Sciences, National Cancer Institute, Bethesda, MD 20892 USA; 4 Department of Pathology and Laboratory Medicine, McGovern Medical School, UT Health Science Center at Houston, Houston, TX 77030 USA

**Keywords:** Cancer, Biomarkers

## Abstract

Hepatocyte nuclear factor 1 homeobox alpha (*HNF1α*) is a transcription factor involved in endodermal organogenesis and pancreatic precursor cell differentiation and development. Earlier studies have reported a role for *HNF1α* in pancreatic ductal adenocarcinoma (PDAC) but it is controversial. The mechanism by which it impacts PDAC is yet to be explored in depth. In this study, using the online databases we observed that *HNF1α* is upregulated in PDAC, which was also confirmed by our immunohistochemical analysis of PDAC tissue microarray. Silencing *HNF1α* reduced the proliferative, migratory, invasive and colony forming capabilities of pancreatic cancer cells. Key markers involved in these processes (pPI3K, pAKT, pERK, Bcl2, Zeb, Snail, Slug) were significantly changed in response to alterations in *HNF1α* expression. On the other hand, overexpression of *HNF1α* did not induce any significant change in the aggressiveness of pancreatic cancer cells. Our results demonstrate that reduced expression of *HNF1α* leads to inhibition of pancreatic cancer growth and progression, which indicates that it could be a potential oncogene and target for PDAC.

## Introduction

Advances in cancer research, diagnosis, and treatment have led to an overall increase in the 5-year survival rate for all cancers combined. However, minimal progress has been made with respect to pancreatic ductal adenocarcinoma (PDAC)—a highly lethal disease with a 5-year survival rate of only 8%^1^. While PDAC is not among the most common forms of cancer, it is predicted to become the second leading cause of cancer-related death by 2030^2^. Given that less than 20% of PDAC patients present as suitable candidates for surgical resection, chemotherapy remains the main mode of treatment^1^. However, chemotherapy is often ineffective, due to the highly metastatic nature and complexity of the disease^3^. While identifying potential biomarkers for development of targeted drug therapy remains critical, there is also a strong need to reassess the role of specific biomarkers that may provide insight into the intricate nature of this disease.

One such biomarker is hepatocyte nuclear factor 1 alpha (*HNF1α*), a transcription factor first identified in hepatocytes that is expressed in the yolk sac endoderm, and in the developing kidney, liver, and pancreas in a spatio-temporal manner^4^. In adult tissues, HNF-1 regulates epithelia-specific gene expression of the gut, lung, liver, pancreas, and urogenital tract^5–7^. Aside from its role in liver development, *HNF1α* has also been shown to be involved in pancreas development as one of the central regulators of pancreatic precursor cell differentiation^8,9^. In some previous studies, PDAC tissue was found to express lower levels of *HNF1α* compared to normal pancreatic tissue, suggesting that its dysregulation may be a key step in PDAC development^9^. As such, *HNF1α* has been suggested to play a tumor suppressing role, specifically in both liver and pancreatic cancers. However, recent studies have shown an oncogenic role for *HNF1α*^10,11^. The current knowledge on the exact role of HNF-1α in pancreatic carcinogenesis is contradictory, and the significance of targeting this protein for therapeutic purposes remains unidentified. Its mechanism of action within PDAC has yet to be elucidated.

With this previous knowledge in mind, we set out to study the effects of *HNF1α* in PDAC. We focused specifically on the role of *HNF1α* in the development and progression of PDAC in hopes to apply this knowledge in the development of more targeted and effective treatments for this disease. We found that silencing *HNF1α* reduced the proliferative, migratory, invasive and colony forming capabilities of pancreatic cancer cells, consistent with previous research that has identified it as a novel oncogene in pancreatic cancer^10^. Thus, continued research into the role of *HNF1α* is worthy of further exploration.

## Results

### *HNF1α* expression in PDAC cells

**Figure 1:**
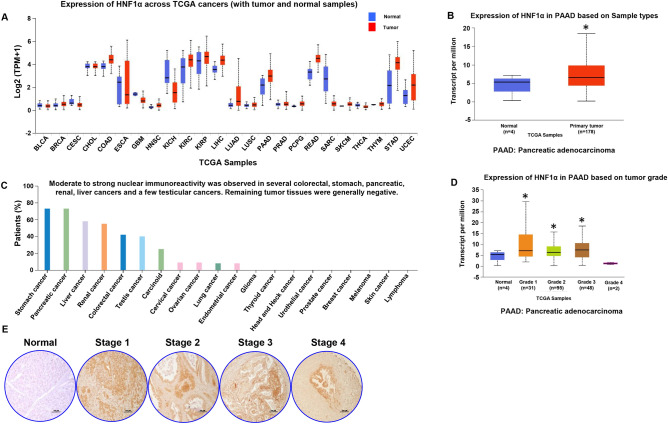
Expression levels of *HNF1α* in cancers. (**A**) Expression of *HNF1α* across TCGA cancers with tumor and normal samples. (**B**) Expression of *HNF1α* in pancreatic adenocarcinoma (n = 178) and normal pancreas (n = 4). (**C**) Immunoreactivity of *HNF1α* across TCGA cancers. (**D**) Expression of *HNF1α* in pancreatic adenocarcinoma based on tumor grade; normal (n = 4), grade 1 (n = 31) grade 2 (n = 95), grade 3 (n = 48) and grade 4 (n = 2). (**E**) Immunohistochemistry analysis of normal pancreas tissue and tumor stages of human pancreatic ductal adenocarcinoma tissues, *HNF1α*-positive cells indicated by brown staining at × 100 magnification. *p < 0.05.

The expression levels of *HNF1α* in all cancers (found by using TCGA database through the UALCAN portal) revealed higher *HNF1α* expression in PDAC pancreata compared to normal pancreata (Fig. 1A,B). Next, we analyzed the immunoreactivity of *HNF1α* among all cancers in the TCGA database and found that PDAC had the second highest immunoreactivity next only to stomach cancer (Fig. 1C). Analysis of expression of *HNF1α* in PDAC based on tumor grade indicates that early stage disease has higher expression and as the disease progresses the expression decreases (Fig. 1D). Immunohistochemical analyses of PDAC TMA revealed increased expression of *HNF1α* in early stage PDAC compared to the normal control, with expression decreasing as the stage of PDAC advanced (Fig. 1E). The difference in staining could be due to the occurrence of huge desmoplasia in advanced pancreatic cancers*.*

**Figure 2:**
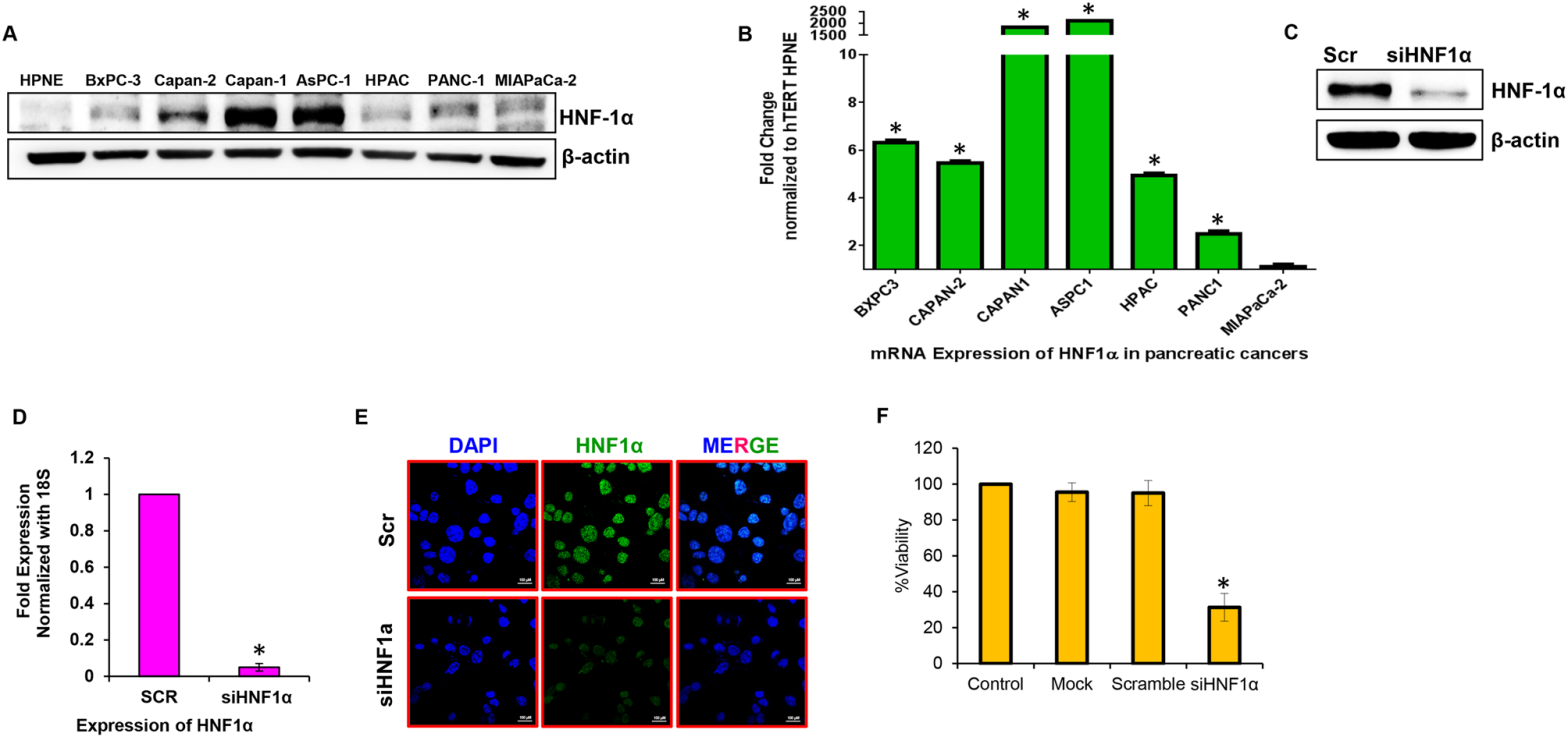
Expression of *HNF1α* in pancreatic cancer lines and its suppression using siRNAs. (**A**) Western blot analysis of *HNF1α* expression in a panel of pancreatic cancer cell lines (HPAC, Capan-2, Capan-1, AsPC-1, PANC-1, Mia PaCa-2, and BxPC-3). (**B**) Real-time PCR analysis of mRNA expression of *HNF1α* in pancreatic cancer lines compared to normal pancreas cell line hTERT HPNE. The fold expression of *HNF1α in* hTERT HPNE was equated to one. Each bar represents the mean of three independent experiments (p < 0.05). Expression of *HNF1α* in hTERT HPNE cells was considered as unit value = 1. (**C**) Western blot analysis of *HNF1α* protein expression in *HNF1α* silenced AsPC-1 pancreatic cancer cells. (**D**) Relative mRNA expression of *HNF1α* was assessed by real-time PCR in si*HNF1α* AsPC-1 cells. Data shown as mean ± SEM. Experiments (n = 3) were repeated three times in triplicates. *p < 0.05. (**E**) Representative photographs of immunofluorescence analysis of *HNF1α* expression in si*HNF1α* AsPC-1 cells at × 100 magnification. (**F**) MTS assay was used to measure cell viability in response to *HNF1α* silencing in AsPC-1 cells.

The expression of *HNF1α* was analyzed in several PDAC cell lines (BxPC-3, Capan-2, Capan-1, AsPC-1, HPAC, PANC-1, and MIAPaCa-2) and normal pancreas cell line (hTERT HPNE). Western blot analysis showed that there was a range of *HNF1α* expression in this panel of pancreatic cell lines, with AsPC-1 having very high *HNF1α* expression and HPAC having very low *HNF1α* expression (Fig. 2A). Data from qRT-PCR analysis was similar to western blot analysis; we observed that the PDAC cells express higher levels of *HNF1α* compared to normal pancreatic cells (Fig. 2B). Immunofluorescence analysis also verified the increased expression levels of *HNF1α* in AsPC-1 cells compared to hTERT HPNE normal pancreas cells, showing *HNF1α* is mainly localized in the nucleus of the PDAC cells (Supplementary Fig. S1A).

To investigate its role in PDAC, *HNF1α* was silenced in AsPC-1 and overexpressed in HPAC cells. Three distinct *HNF1α*-specific siRNAs (subtypes A, B, and C) were used to observe the efficacy of silencing *HNF1α*. Western blot analysis revealed the 30 nM siRNA subtype “A” as most effective in silencing *HNF1α* in AsPC-1 cells (Fig. 2C and Supplementary Fig. S1B), which was further validated by qRTPCR (Fig. 2D) and immunoflurorescence (Fig. 2E). Three different colonies transfected with 1 µg concentration of *HNF1α* pcDNA were used to survey the efficacy of *HNF1α* overexpression. Colony 3, which was transfected with 1 µg of *HNF1α* pcDNA, showed the highest expression of *HNF1α* according to western blot analysis (Supplementary Fig. S2A). qRT-PCR analysis performed confirmed the efficacy of overexpression of *HNF1α* in HPAC cells (Supplementary Fig. S2B). The *HNF1α*-silenced AsPC-1 and *HNF1α*-overexpressing HPAC cells generated were used for all further experiments.

### *HNF1α* influences cell proliferation and survival in PDAC cells

Cancer cells retain the ability to proliferate rapidly and evade death^12^. In order to assess whether *HNF1α* expression influences these abilities in pancreatic cancer cells, cell viability was measured in *HNF1α*-silenced AsPC-1 cells and *HNF1α*-overexpressing HPAC cells. Silencing *HNF1α* decreased the viability of AsPC-1 cells significantly (by nearly 70%) when compared to scramble control AsPC-1 cells (Fig. 2F). Overexpression of *HNF1α*, on the other hand, had a very minimal effect on the viability of HPAC cells when compared to pCMV control HPAC cells (Supplementary Fig. S3A).

### Targeting *HNF1α* impacts proliferation of PDAC cells through PI3K-AKT-mTOR signaling

**Figure 3:**
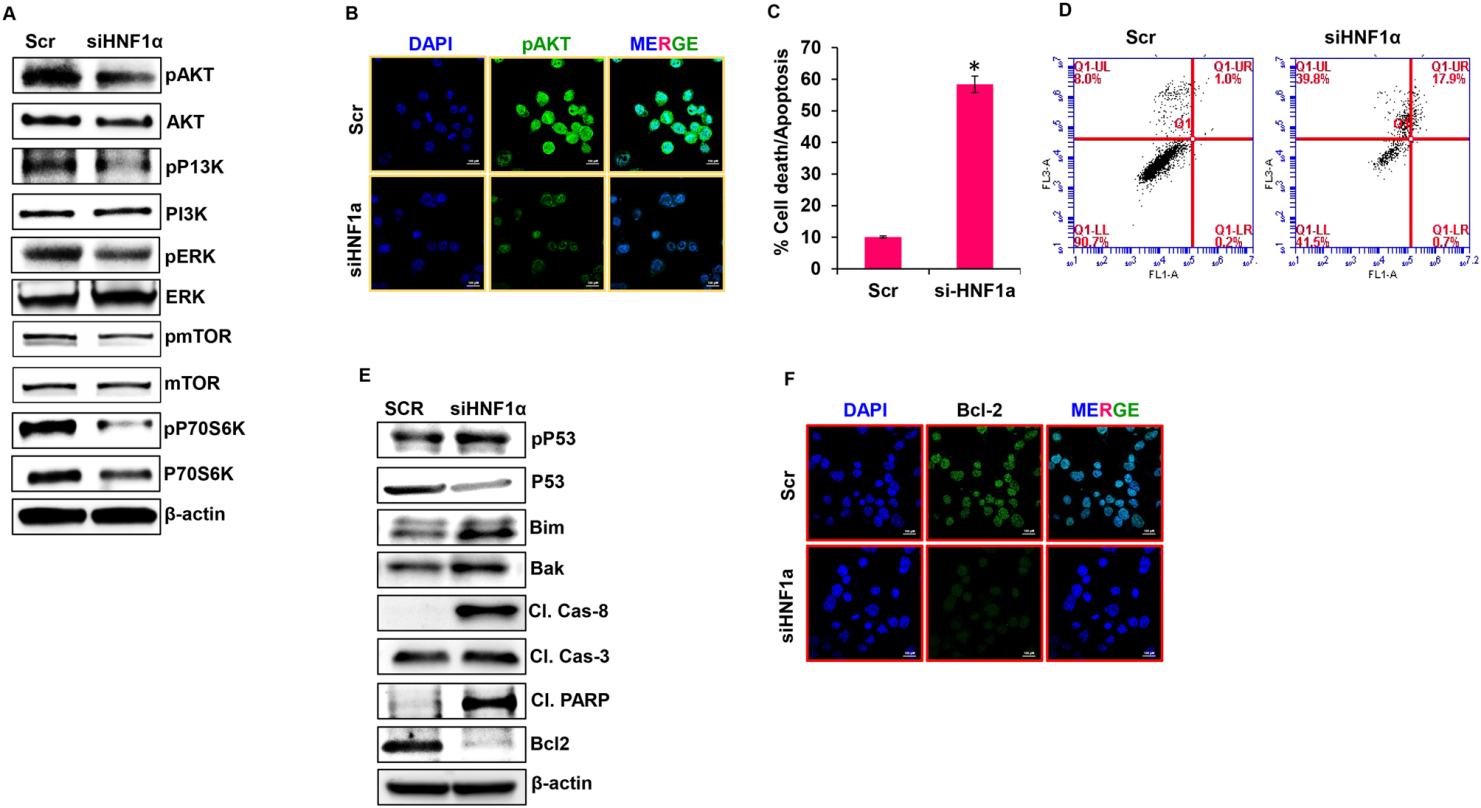
Silencing *HNF1α* gene expression inhibits proliferation and induces apoptosis in PDAC. (**A**) Expression levels of pAKT, AKT, PP13K, PI3K, pERK, ERK, pmTOR, mTOR, pP70S6K, and P70S6K were determined by western blot in silenced *HNF1α* AsPC-1 cells. (**B**) Immunofluorescence analysis showed cell proliferation markers for pAKT in si*HNF1α* AsPC-1 cells at × 100 magnification. (**C**,**D**) Apoptosis analysis of si*HNF1α* AsPC-1 cells measured by flow cytometry. (**E**) Western blot analysis of apoptotic markers in si*HNF1α* AsPC-1 cells. (**F**) Immunofluorescence analysis of BCl2 in si*HNF1α* AsPC-1 cells at × 100 magnification. Data shown as mean ± SEM. Experiments (n = 3) were repeated three times in triplicates. *p < 0.05.

Silencing *HNF1α* in AsPC-1 cells significantly reduced expression levels of the active forms of AKT, PI3K, ERK, mTOR and p70s6k. Accumulation of total forms of ERK, mTOR and p70S6K was observed in *HNF1α* silenced AsPC-1 cells (Fig. 3A). Overexpression of *HNF1α* in HPAC did not significantly reduce the active forms of these proteins (Supplementary Fig. S3B). Immunofluorescence imaging also verified the reduced expression of proliferative marker pAKT in response to suppression of *HNF1α* (Fig. 3B). These data reveal that silencing *HNF1α* alters the proliferative and survival potential of PDAC cells through the PI3K/AKT/mTOR signaling pathway.

### Suppressing *HNF1α* gene expression induces apoptosis

The role of *HNF1α* in apoptosis was examined using Annexin V/PI staining. Silencing *HNF1α* drastically increased apoptosis by 48.3% (Fig. 3C,D) while over expression of *HNF1α* slightly reduced apoptosis by 3.2% (Supplementary Fig. S4A & S4B). To further confirm the role of *HNF1α* in apoptosis, the key molecular markers involved in this process were studied. Silencing *HNF1α* in AsPC-1 cells resulted in increased levels of pro-apoptotic proteins p53, Bim, Bak, cleaved caspases 3 and 8, and cleaved PARP, while the expression of anti-apoptotic protein Bcl-2 was reduced (Fig. 3E). Over expression of *HNF1α* in HPAC cells did not change these apoptotic markers (Supplementary Fig. S4C). Immunofluorescence data further confirmed the role of *HNF1α* in apoptosis by detecting the reduced expression of anti-apoptotic marker Bcl-2 in response to suppression of *HNF1α* (Fig. 3F). Our results indicate that the significance of *HNF1α* expression is crucial in apoptosis.

### *HNF1α* influences migration, invasion, and anchorage-independent growth of PDAC cells

**Figure 4:**
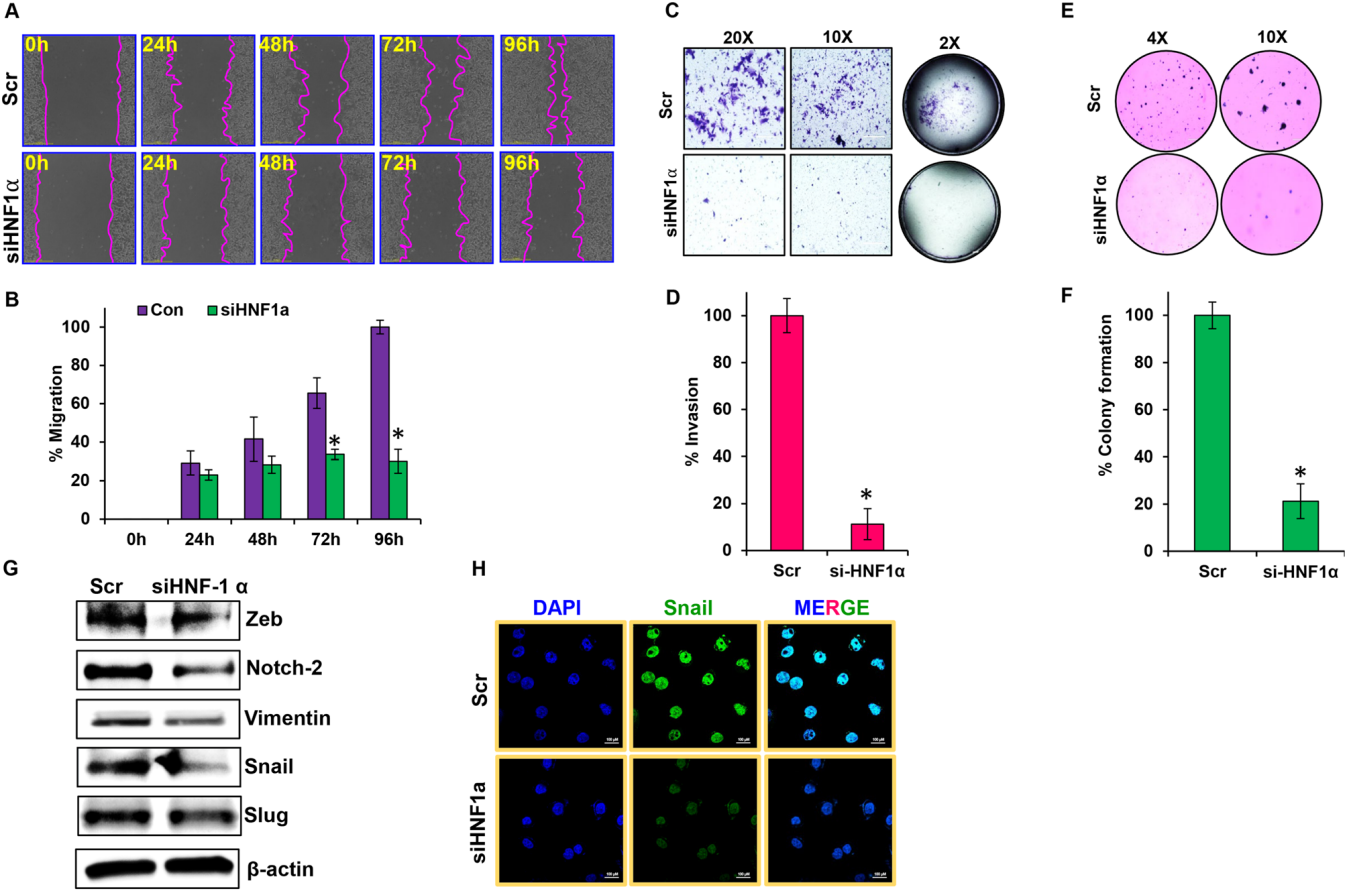
*HNF1α* regulates metastatic characteristics of PDAC. (**A**,**B**) Wound-healing assay was performed in si*HNF1α* AsPC-1 cells; migration was analyzed using Nikon Biostation CT at 2 h intervals for up to 96 h at × 4 magnification. (**C**,**D**) Invasiveness of si*HNF1α* AsPC-1 cells and overexpression *HNF1α* HPAC cells were observed using a Matrigel invasion assay, and captured using Nikon Eclipse TS 100 microscope at × 20 and × 100 magnification. (**E**,**F**) Colony formation assay was performed in si*HNF1α* AsPC-1 cells. (**G**) EMT markers analyzed by western blot in si*HNF1α* AsPC-1 cells. (**H**) Immunofluorescence analysis of Snail in si*HNF1α* AsPC-1 cells, captured using Nikon SMZ 1500 microscope at × 40 magnification. Data shown as mean ± SEM. Experiments (n = 3) were repeated three times in triplicates. *p < 0.05.

Silencing *HNF1α* in AsPC-1 cells significantly decreased the migratory abilities of these cells when compared to the control cells (Fig. 4A). Control cells migrated nearly 100% and closed the scratch within 96 h, while *HNF1α*-silenced AsPC-1 cells only migrated about 30% (Fig. 4B). Overexpression of *HNF1α* in HPAC cells had no observable effect on the migratory abilities of these cells compared to control cells (Supplementary Fig. S5A,B). Silencing *HNF1α* reduced invasion by nearly 90% in AsPC-1 cells compared to the control (Fig. 4C,D). Overexpression of *HNF1α*, however, had very little effect (< 20%) on the invasive capabilities of HPAC cells compared to the control (Supplementary Fig. S5C,D).

Silencing *HNF1α* greatly reduced colony formation (~ 80%) in AsPC-1 cells compared to the control cells (Fig. 4E,F). There was no significant effect on colony formation when *HNF1α* was overexpressed in HPAC cells compared to the control cells (Supplementary Fig. S6A,B). Overall, the data collected suggest that silencing *HNF1α* greatly reduces migration, invasion, and colony formation in PDAC cells.

To confirm *HNF1α*’s contribution in metastatic colony formation, we studied a variety of key genes that have been shown to be involved in epithelial to mesenchymal transition (EMT) using western blotting analysis. Suppressing *HNF1α* expression in AsPC-1 cells, resulted in an observable reduction in Zeb, Notch-2, Vimentin, Snail, and Slug expression (Fig. 4G), while overexpressing *HNF1α* in HPAC was not able to effect any change in the expression of these EMT markers (Supplementary Fig. S6C). Immunofluorescence imaging also verified the reduced expression levels of Snail in response to suppression of *HNF1α* in PDAC cells (Fig. 4H). These data indicates that *HNF1α* influences metastatic processes by altering the expression of key signaling molecules involved in EMT.

### Impact of *HNF1α* expression in PDAC preclinical models

**Figure 5:**
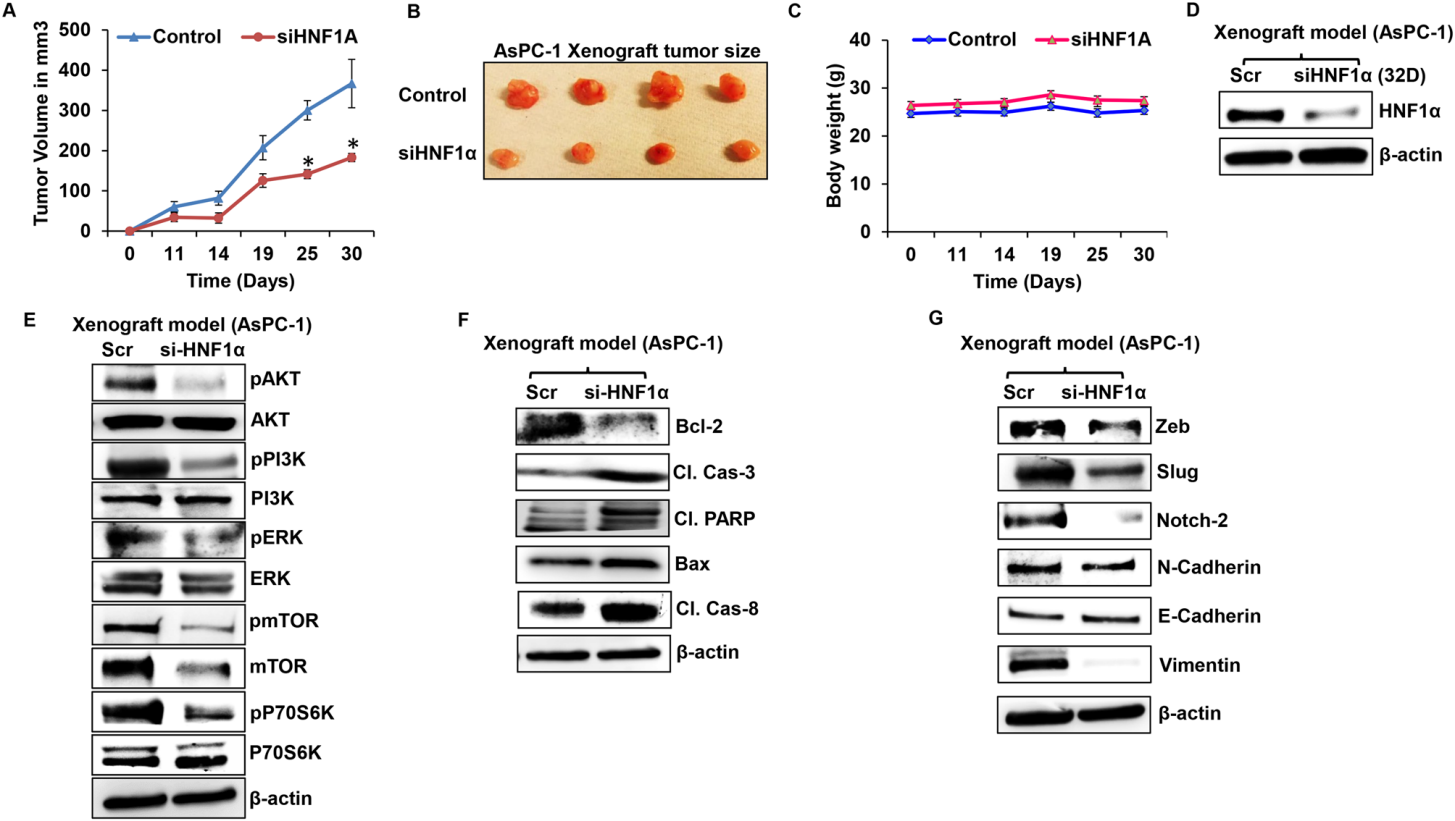
Silencing *HNF1α* inhibits PDAC tumorigenesis. (**A**) Tumor growth curve of si*HNF1α* AsPC-1 cells. (**B**) AsPC-1 Xenograft tumors from control and si*HNF1α* silenced groups (n = 6). (**C**) Body weights of AsPC-1 control and si*HNF1α* silenced experimental nude mice (n = 6). (**D**) Western blot analysis of *HNF1α* protein expression in si*HNF1α* AsPC-1 cells. (**E**) Western blot analysis of cell proliferation markers in si*HNF1α* AsPC-1 xenografts. (**F**) Western blot analysis of apoptotic marker in si*HNF1α* AsPC-1 cells and overexpression *HNF1α* in HPAC xenograft. (**G**) Western blot analysis of EMT markers in si*HNF1α* AsPC-1 cells. Data shown as mean ± SEM. Experiments (n = 3) were repeated three times in triplicates. *p < 0.05.

To confirm our in vitro outcomes, we also conducted in vivo studies using athymic nude mice. In these mice, either *HNF1α* silenced AsPC-1 cells or parental AsPC-1 cells were transplanted into the flanks of the nude mice. By silencing *HNF1α*, tumor growth was reduced in xenografts when compared to control tumors (Fig. 5A,B). There was not significant change in the body weights among the two groups (Fig. 5C). The xenograft tumors from both the groups were surgically excised and used for molecular analysis. First, we looked for the levels of expression of *HNF1α* and found that *HNF1α* silenced xenografts still had lower levels of *HNF1α* expression compared to control xenografts (Fig. 5D).

Further, we examined effect of *HNF1α* on proliferation, EMT, and apoptosis markers using xenograft tumor tissues from *HNF1α* silenced AsPC-1 and control xenograft tumor tissues. Suppression of *HNF1α* led to downregulation of proliferative markers: AKT, PI3K, ERK, mTOR and P70S6K (Fig. 5E). Apoptotic markers were analyzed in the *HNF1α*-silenced group and showed decreased anti-apoptotic marker Bcl-2, increased cleaved caspase 3, and cleaved PARP. Bax and caspase 8 were decreased significantly compared to control group (Fig. 5F). Immunoblot data showed a decrease in EMT markers in *HNF1α*-silenced tissue (Zeb, Slug, Notch-2, N-Cadherin, and Vimentin), while E-cadherin levels stayed constant. EMT markers Snail and N-Cadherin were both downregulated in the *HNF1α*-silenced AsPC-1 xenograft tumor tissues compared to the control experimental group (Fig. 5G).

## Discussion

Initially *HNF1α* was described as a glucose metabolism regulator, with a possibly important role in diabetes^13–15^. *HNF1α* is also potentially enriched in the exocrine-like/ADEX group of tumors, and the *HNF1α* positive subtype of PDAC is resistant to certain tyrosine kinase inhibitors and paclitaxel^16^. *HNF1α* -positive PDAC patients are expected to respond better to intensive chemotherapy according to the FOLFIRINOX protocol^17^.

The data from TCGA database analyzed showed that the expression and immunoreactivity of *HNF1α* was upregulated in PDAC tissues compared to normal tissues. Further, the dataset also showed the differential expression of *HNF1α* at different PDAC tumor grades. Immunohistochemical analysis of the PDAC tissue microarray also showed that the expression of *HNF1α* protein was higher in PDAC tissues compared to normal noncancerous tissues. Further, it also confirmed the differential expression of *HNF1α* at different tumor grades.

We found high expression of *HNF1α* in PDAC cell lines compared to the normal pancreas cell line. In particular, the expression of *HNF1α* is high in AsPC-1 and Capan-1 compared to other panels of PDAC cell lines screened. This could be due to the fact that these two cell lines were derived from metastatic sites, while the other cell lines were derived from primary cancers. In addition, it has been demonstrated that the doubling time of AsPC -1 cells is shorter than the other PDAC cell lines^18^. This finding provides compelling insight that *HNF1α* may play a role in PDAC. When examining the role of *HNF1α* on PDAC cells after altering its expression, the present study found that *HNF1α* is an oncogene. For a gene to be considered as an oncogene, it has to have the ability to initiate and promote cancer development^19^. In this process, it is well established that epithelial to mesenchymal transition plays a vital role in inducing and increasing cancer cell invasion, migration and colony forming capabilities^20,21^. Decreased expression of *HNF1α* resulted in significantly reduced levels of proliferation, migration, invasion, and colony formation of PDAC cells, compared to the control. On the other hand, when overexpressed, *HNF1α* had little to no effect on these same PDAC cell abilities. Thus, these findings suggest *HNF1α* plays a role as an oncogene and not as a tumor suppressor, as previously thought.

Earlier it has been demonstrated that tumor suppressor miR-484 modulates the ZEB1 and SMAD2^22^ and WNT/ MAPK pathway by directly targeting *HNF1α* in cervical cancer cells^11^. Further, it also been shown that *HNF1α* significantly promoted pancreatic cancers by influencing fibroblast growth factor receptor 4^23^. Recently, it has been shown that *HNF1α* is a putative regulator of PDAC stem cell gene signature^10^. Further, it has also been demonstrated that *HNF1α* is required for tumor growth, tumorsphere formation, invasion and migration. In addition, the mechanism by which *HNF1α* promotes PDAC stemness is by regulating the pluripotency factor POU5F1/OCT4. Expression of *HNF1α* upregulated genes has been associated with poor survival outcomes in PDAC patients^10^.

Our data demonstrates that overexpression of *HNF1α* did not significantly alter any of the key processes and key markers involved in PDAC growth and progression. The reason we did not see any significant effect of *HNF1α* overexpression on PDAC growth and progression could be due to the fact that HPAC cells are highly aggressive to begin with, and increasing the expression of *HNF1α* might not have been able to increase their aggressiveness any further.

Overall, our data demonstrate that when silenced, *HNF1α* significantly reduces PDAC cell proliferation, migration, invasion, and colony formation, demonstrating the oncogenic role it plays in PDAC. While the exact role of *HNF1α* remains somewhat elusive, it provides insight into the complexity of PDAC and demonstrates a need for further studies that may lead to the identification of suitable targets for successful PDAC treatment. Considering the highly deadly and intricate nature of this disease, it is critical that targets of interest be evaluated completely in order to establish successful PDAC treatments that may change the dire outlook for patients with this disease.

## Materials and methods

### Cell lines

HPAC, Capan-2, Capan-1, AsPC-1, PANC-1, MIA PaCa-2 and BxPC-3 (pancreatic cancer cell lines) and hTERT HPNE (normal pancreas cell line) were all obtained from the American Type Culture Collection. All cell lines were tested for mycoplasma contamination. HPAC, PANC-1, BxPC-3 and AsPC-1 cell lines were cultured in RPMI-1640 media containing 10% FBS, 100 Units/mL of penicillin, and 100 μg/mL of streptomycin. All cells were cultured as described in our earlier study^24^.

### UALCAN database analysis

UALCAN (https://ualcan.path.uab.edu) is a publically available database, which is a comprehensive and interactive online resource that can be used to analyze cancer omics data. This web resource is built on PERL-CGI platform. This web resource provides access to TCGA datasets and also provides additional information about genes by linking to the human protein atlas, Pubmed, Human Protein Resource Database, GeneCards, etc.^25^. We used the UALCAN database to identify the significance of *HNF1α* expression in pancreatic cancer. We analyzed the expression and immunoreactivity of *HNF1α* across TCGA cancers including pancreatic cancers. We further also analyzed the expression of *HNF1α* in pancreatic cancer based on tumor grade.

### Silencing of *HNF1α* in AsPC-1 Cell Line

AsPC-1 cells were seeded in a 6-well plate at a density of 2.5 × 10^5^ cells/well. Twenty-four hours after seeding, cells were transfected with various concentrations (10 nM, 30 nM and 50 nM) of *HNF1α* different siRNAs (subtype A, B and C) for 48 h (Origene, Catalog # SR304741). Scrambled siRNA was used as a control. MIrus bio TransIT siQUEST transfection reagent (Mirus Bio) was used to perform all the siRNA transfection studies as described earlier^24,26^.

### Sequences for HNF1α siRNA subtypes “A”, “B”, and “C”

SR304741A-rGrGrArGrUrGrCrArArUrArGrGrGrCrGrGrArArUrGrCrATC.

SR304741B-rGrGrArCrArGrGrArCrUrArArCrArCrUrCrArGrArArGrCCT.

SR304741C-rCrGrGrUrGrUrGrCrGrCrUrArUrGrGrArCrArGrCrCrUrGCG.

### Overexpression of *HNF1α* in HPAC Cells

DH5α competent cells (Invitrogen) were transformed with 2 µg of plasmid DNA for human *HNF1α* (Origene, Catalog # SC300093) or PCMV-XL6 cloning vector alone (Origene, Catalog # PCMV6XL6). The transformed cultures were spread on a pre-warmed 4% agar plate with 100 µg/mL ampicillin (Life Technologies). The plate was incubated at 37 °C overnight. Single colonies were selected and inoculated in LB broth with 25 µg/mL ampicillin overnight. DNA was isolated and purified from *HNF1α* plasmid-transfected E.coli cells using Invitrogen Pure Link HiPure Plasmid Filter Maxiprep Kit (Life Technologies). HPAC cells were seeded in 6-well plates at a density of 2.5 × 10^5^ cells/well. Twenty-four hours after seeding, cells were transfected with 1 μg of *HNF1α* pDNA from four different colonies. The transfection was carried out using MIrus bio TransIT 2020 transfection reagent (Mirus Bio) as described earlier^24^. In order to maintain efficient overexpression and avoid toxicity, the ratio of transfection reagent to plasmid DNA was conserved at 5:1 according to the manufacturer’s protocol. *HNF1α* overexpression was verified with western blot and immunofluorescence analysis. The third colony transfected with 1 μg *HNF1α* pDNA was the most effective in overexpressing *HNF1α*. This was used for all *HNF1α* overexpression experiments, including cell proliferation, migration, invasion, colony formation assay, immunofluorescence, xenograft studies, mRNA, and protein analysis.

### Pancreatic adenocarcinoma tissue array

The pancreatic adenocarcinoma tissue microarray (TMA) from US Biomax, Inc. were subjected to immunohistochemistry (IHC) to measure *HNF1α* expression levels. This microarray consist of formalin-fixed paraffin embedded samples (n = 24) of pancreatic adenocarcinoma at different stages along with normal pancreatic tissues.

### Immunohistochemical analysis (IHC)

IHC was performed as described earlier^27^. In brief, TMA sections were deparaffinized and hydrated using decreasing concentrations of ethanol baths. Following antigen retrieval with trilogy, the tissue samples were blocked and then incubated at 4 °C with a primary antibody against *HNF1α* followed by Ultra Marque polyscan HRP labeled secondary antibody (Cell Marque). Sections were stained with 3,3′diaminobenzidine and counterstained with hematoxylin and dehydrated. Finally, the slides were sealed with mounting media (Surgipath Medical Industries), and images were captured using a Nikon Microscope-ECLIPSE 50i. IHC staining for different proteins were quantitated at 5 random areas per section with at least 200 cells per field.

### Cell viability assay

The MTS (3-(4,5-dimethylthiazol-2-yl)-5-(3-carboxymethoxyphenyl)-2-(4-sulfophenyl)-2H-tetrazolium) assay was performed as described earlier^28^. MTS reagent was added for 4 h to *HNF1α*-silenced AsPC-1 cells and *HNF1α*-overexpressing HPAC cells t and cell viability was measured using a microplate reader (CLARIOstar, BMG LABTECH).

### Wound healing assay

*HNF1α*-silenced AsPC-1 cells and *HNF1α*-overexpressing HPAC cells, were seeded in 6-well plates at a density of 2.5 × 10^5^ cells/well and maintained at 37ºC in a 5% CO_2_ environment for 48 h. A scratch was created with a sterile pipette tip once the cells reached monolayer confluency. The cells detached due to the scratch were removed and the respective complete growth media was added to the plates. Then, the cells were provided their respective complete growth media. The distance migrated by the cells were capturedand calculated at 2 h intervals for 96 h using the Nikon Biostation CT. NIS-Element AR software^26^.

### Matrigel invasion assay

*HNF1α*-silenced AsPC-1 cells and *HNF1α*-overexpressing HPAC cells were plated in the upper chamber of a transwell polycarbonate insert coated with matrigel (1 mg/ml) at a density of 8 × 10^4^ cells/well. The complete growth media for each cell line was added as a chemoattractant in the lower chamber of the transwell. The invaded cells were fixed and stained with 0.2% crystal violet in 5% formalin. Excess stain was removed by washing with PBS. Nikon Eclipse TS 100 microscope was used to capture five arbitrarily selected fields to calculate the number of invaded cells^26^.

### Colony formation assay

A clonogenic assay was also performed with si*HNF1α* AsPC-1 cells and OV*HNF1α* HPAC cells, each plated separately on 60-mm dishes with a top layer of 0.7% agarose at a density of 2 × 10^4^ cells and a bottom layer of 1% agar. Cells were maintained by changing the complete growth media three times a week. Then, colonies were fixed and stained with 0.2% crystal violet in 5% formalin solution. Colonies were counted manually and images were obtained using a Nikon SMZ 1500 microscope as described earlier^27^.

### Immunofluorescence analysis

AsPC-1 and HPAC cells were each plated at a density of 10 × 10^3^ cells/well in an 8-well chamber slide. Twenty-four hours after seeding, the AsPC-1 and HPAC cells were transfected with *HNF1α* siRNA or *HNF1α* overexpression plasmid and incubated for 48 h. The cells were fixed with 100% methanol for 10 min, followed by 100% acetone fixation for another 10 min. Cells were permeabilized using 0.2% Triton X-100 in PBS for 20 min and blocked with 5% BSA for 1 h. Slides were incubated overnight with the primary antibody. Subsequently, Alexa fluor 488-conjugated secondary antibody (Life Technologies) was added and incubated for 1 h. The cells were then washed and counterstained with DAPI. Slides were observed using a Nikon laser scanning confocal microscope.

### Quantitative reverse transcriptase real time PCR (qRT-PCR)

Total RNA was extracted from hTERT HPNE and PDAC cell lines using the Trizol reagent (Invitrogen, Carlsbad, CA, USA). RNA concentration and quality were verified using a NanoDrop 2000 spectrophotometer (ThermoFisher Scientific). cDNA was prepared using the RT2 first strand kit (Qiagen). Human specific primers for *HNF1α* (QT00085428) and RRN18S (QT00199367) uwere purchased from Qiagen. The qRT-PCR was performed in triplicates using the Quantitech SYBR green kit (Qiagen) in a StepOne Plus Real Time PCR system (Applied Biosystems). The data from qRT-PCR was analyzed using comparative Ct method as described earlier^24^.

### Western blot analysis

Mammalian protein extraction reagent (M-PER) was used to extract proteins from cultured cells and xenograft tumor tissues. BCA method was used to quantify the extracted proteins at 490 nm using withCLARIOstar, BMG LABTECH. Proteins were resolved using SDS-PAGE and then transferred onto PVDF membranes. The membranes were blocked with 5% BSA (Sigma-Aldrich Corporation), for 45 min. The membranes were incubated overnight at 4ºC with primary antibodies. The membranes were washed 3 times with TBS-T, followed by a 2-h incubation with the respective secondary horseradish peroxide-coupled antibodies. The expression levels of proteins from various treated groups were measured using enhanced chemiluminescence (GE Las-4000) as described earlier^29^.

All methods described above were in accordance to the institutional guidelines and approved by Texas Tech University Health Sciences Center El Paso Institutional Biosafety Committee.

### Xenograft studies

All the animal experiments performed in this study followed the institutional guidelines and was approved by the Texas Tech University Health Sciences Center El Paso Institutional Animal Care and Use Committee. HNF1α silenced AsPC-1 cells and their respective parental cells, were implanted subcutaneously in both the flanks (1.0 × 10^6^ cells/flank) of six week old male athymic nude mice (Harlan Laboratories) (*n* = 6). Tumor volume and body weight were measured twice a week. Thirty days post transplantation, the mice were euthanized and the xenograft tumors were surgically excised. Excised tissues were fixed in formalin or snap frozen for further histological and molecular analysis respectively^24^.

### Statistical analysis

Statistical analysis was performed using GraphPrism version 6.01 software (GraphPad Software, Inc.). Power analysis was used to calculate sample size and to study the effect difference between groups. Statistical significance was calculated by two-tailed unpaired *t*-test on two groups. A value of *p* < 0.05 was considered statistically significant. All data are expressed as mean ± SEM from at least three independent experiments. All animal experiments had at least 6 animals per group. Animals were randomly assigned to the different groups.

## Supplementary information

Supplementary Information.
