# Supplementary material for: Hepatocyte nuclear factor 1 alpha influences pancreatic cancer growth and metastasis
Source: Sci Rep. 2020 Nov 19;10:20225. doi: 10.1038/s41598-020-77287-5 (PMC7678871; doi:10.1038/s41598-020-77287-5)

Hepatocyte nuclear factor 1 alpha influences pancreatic cancer growth and metastasis

Ramadevi Subramani[1,2*], Ph.D., Joshua Medel[2,3], MS., Kristina Flores[2], BFA., Courtney Perry[2], BS., Adriana Galvez[1], MS., Mayra Sandoval[2], BS., Servando Rivera[2], MS., Diego A. Pedroza[2], MS., Elizabeth Penner[2,4], MS., Mahika Chitti[2] and Rajkumar Lakshmanaswamy[1,2*] Ph.D.

[1] Center of Emphasis in Cancer Research, Department of Molecular and Translational Medicine, Texas Tech University Health Sciences Center El Paso, Paul L. Foster School of Medicine, El Paso, Texas-79905.

[2] Graduate School of Biomedical Sciences, Texas Tech University Health Sciences Center, El Paso, Texas-79905.

[3] Division of Cancer Control and Population Sciences, National Cancer Institute, Bethesda, MD 20892

[4] Department of Pathology and Laboratory Medicine, McGovern Medical School, UT Health Science Center at Houston, Houston, TX 77030

[*]Corresponding Authors

Rajkumar Lakshmanaswamy, PhD

Tel: +1-915-215-4227, Fax: +1-915-783-5222. E-mail: rajkumar.lakshmanaswamy@ttuhsc.edu

Ramadevi Subramani, PhD

Tel: +1-915-215-6851, Fax: +1-915-783-5222. E-mail: ramadevi.subramani@tuhsc.edu

Supplementary materials and methods:

Antibodies and Reagents

The primary antibodies obtained and used for the experiments in this study were: *HNF1α* (12425), Notch 2 (4530), Snail (3879), Zeb (3396), Vimentin (5741), Slug (9585), N-Cadherin (4016), E-Cadherin (3195), pPI3K (4228), PI3K (4292), pmTOR (2974), mTOR (4517), pp70S6 kinase (9206), p70S6 kinase (9202), and Bim (2933), Bak (3814), Bax (2772), Cleaved Caspase 3 (9661), P53, pP53 (Cell Signaling Technology, Boston, MA,USA); Cleaved PARP (sc-8007), pAKT (sc-101629), AKT (sc-5298), pERK (sc-101760), and ERK (sc-94) (Santa Cruz, CA, USA); Caspase 8 (ab61755) and Bcl-2 (ab73985) (Abcam, Cambridge, MA, USA); β-actin (Sigma Aldrich, St. Louis, MO, USA). Respective anti-mouse and anti-rabbit secondary antibodies were acquired from Santa Cruz Biotechnology (Santa Cruz, CA, USA). The Transwell (6.5 mm) with 8.0 μm pore polycarbonate membrane inserts were purchased from Corning Incorporated (Corning, NY, USA). Both BD Matrigel (Bedford, MA, USA) and BD Pharmingen Annexin V-FITC Apoptosis Detection Kit I were obtained from BD Biosciences (San Diego, CA, USA). MTS reagent [3-(4, 5-dimethylthiazol-2-yl)-5-(3-carboxymethoxyphenyl)-2-(4-sulfophenyl)-2H-tetrazolium] was purchased from Promega (Madison, WI, USA). Mammalian protein extraction reagent (mPER) was acquired from Thermo Scientific (Rockford, IL, USA).

Sequences for *HNF1α* siRNA subtypes "A", "B", and "C"

SR304741A-rGrGrArGrUrGrCrArArUrArGrGrGrCrGrGrArArUrGrCrATC

SR304741B-rGrGrArCrArGrGrArCrUrArArCrArCrUrCrArGrArArGrCCT

SR304741C-rCrGrGrUrGrUrGrCrGrCrUrArUrGrGrArCrArGrCrCrUrGCG

Supplementary Figure S1

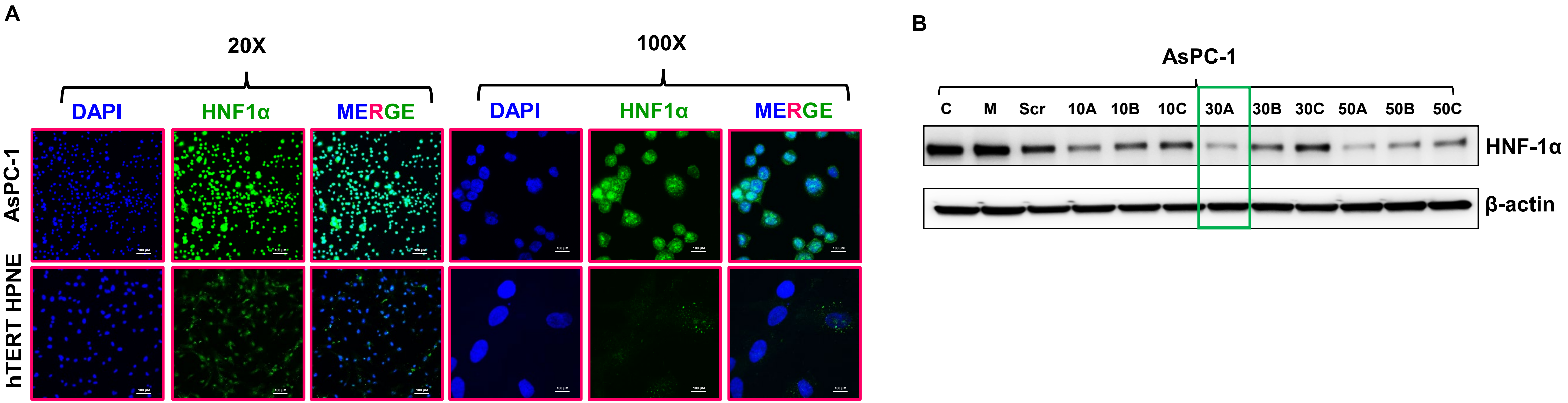

Supplementary Figure S2

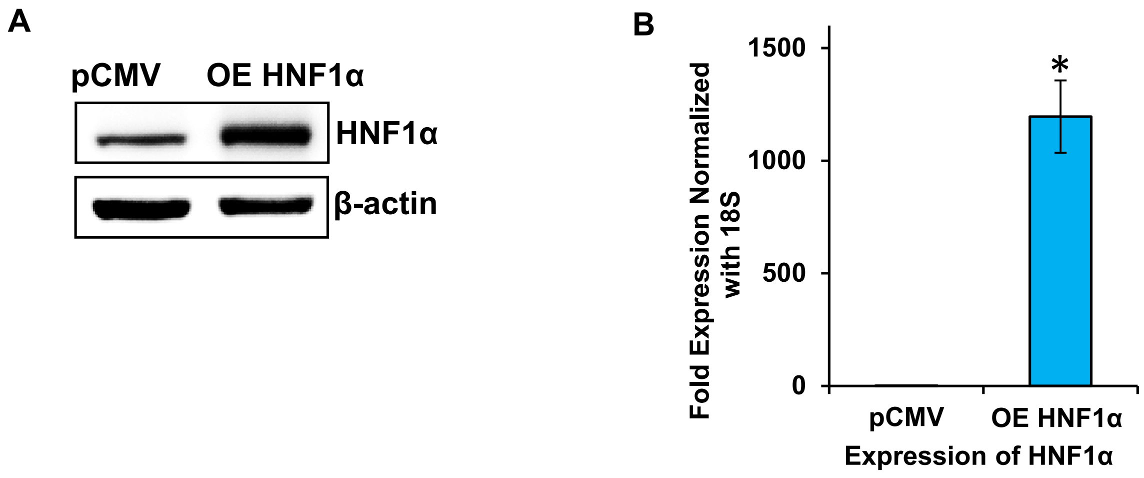

Supplementary Figure S3

A

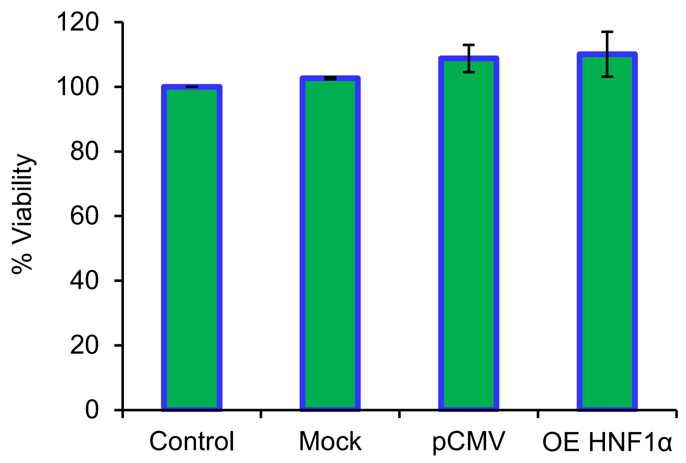

B

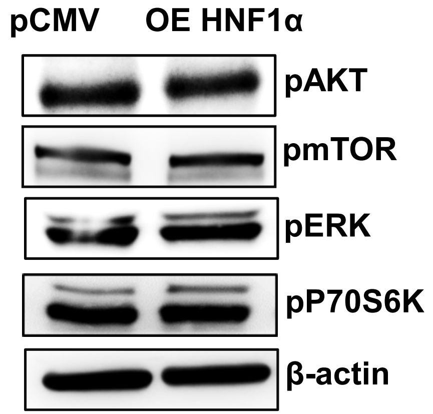

Supplementary Figure S4

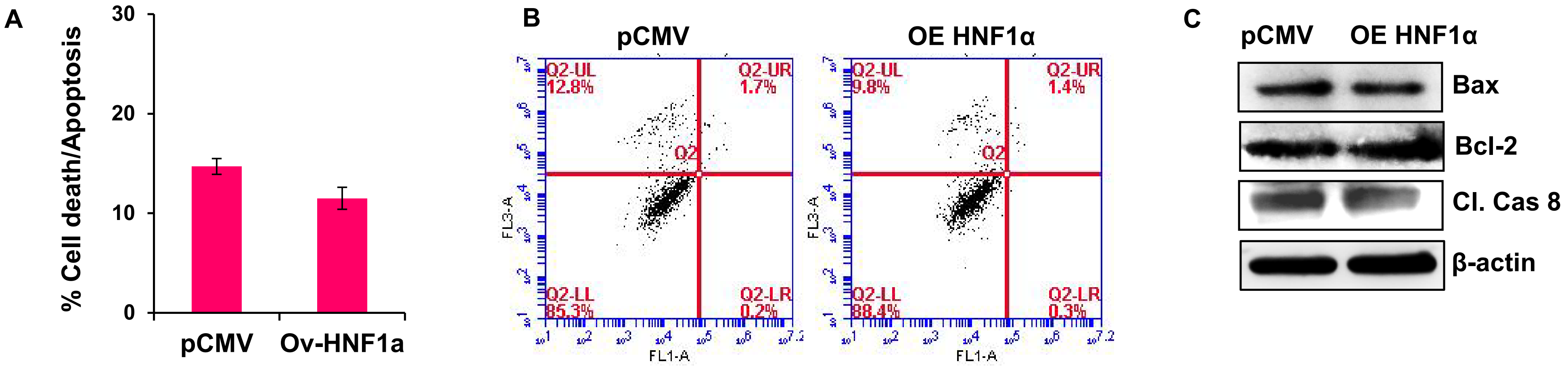

Supplementary Figure S5

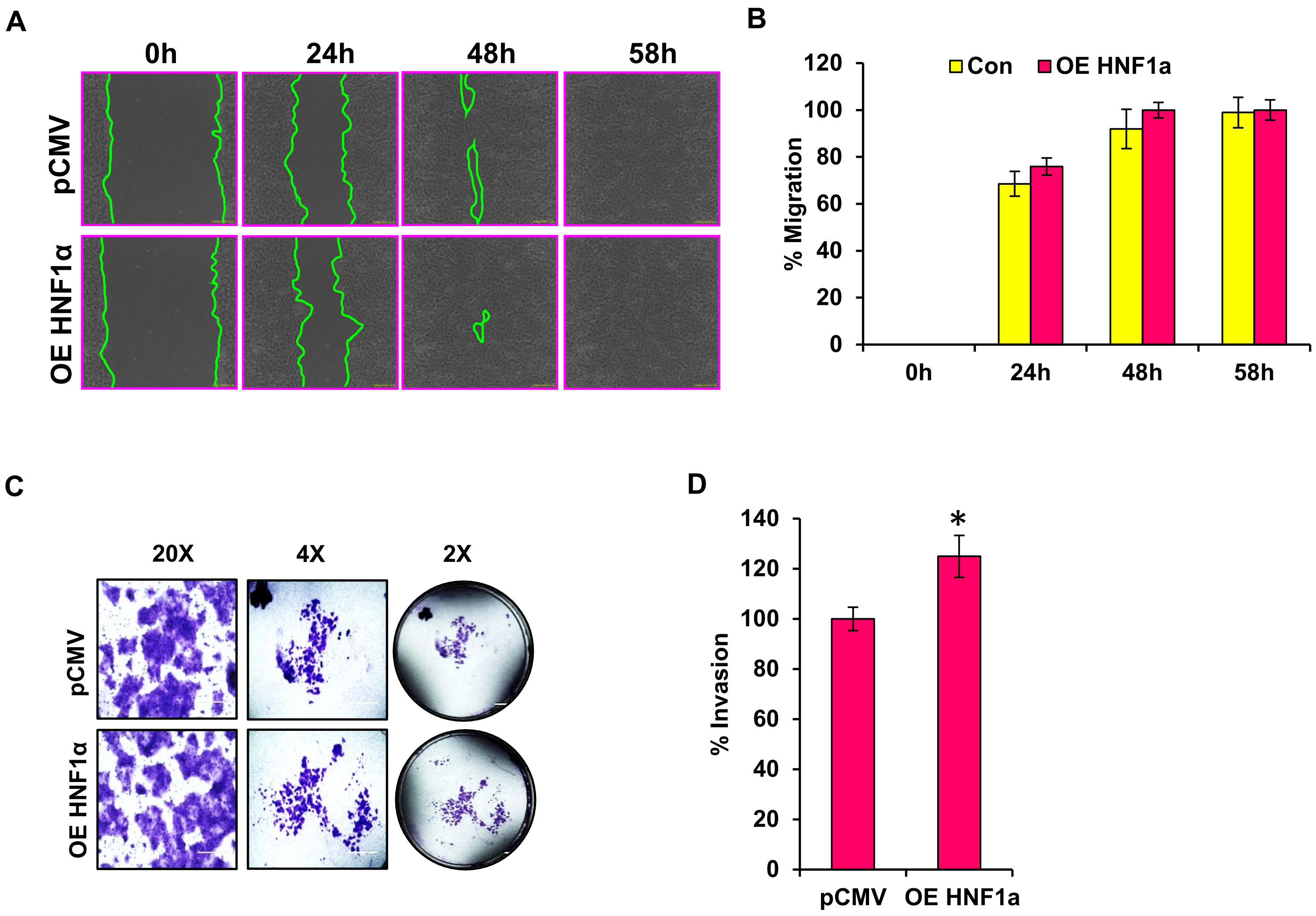

Supplementary Figure S6

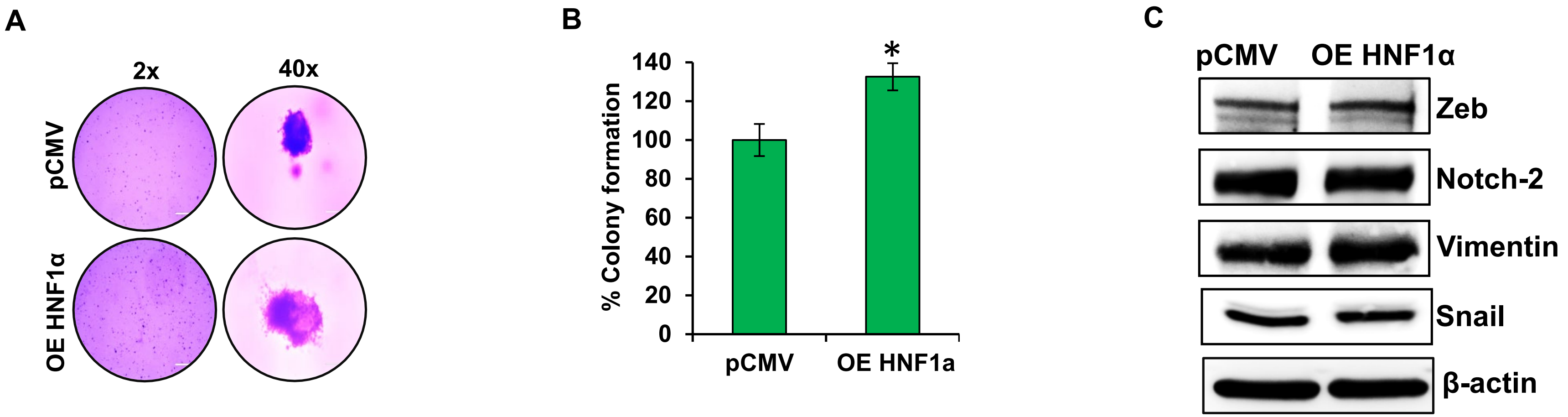

Supplementary Figure Legends:

Supplementary Fig. S1

A. Immunofluorescence of HNF1α expression in AsPC-1 and hTERT HPNE cells (20X and 100X magnification). **B.** AsPC1 cells were transfected with three different predesigned HNF1α siRNAS at different concentrations.

Supplementary Fig. S2

A. Western blot analysis of HNF1α protein expression in overexpressed HNF1α HPAC pancreatic cancer cells. **B.** Relative mRNA expression level of HNF1α in overexpressing HNF1α HPAC cells. Data shown as mean +/- SEM. Experiments (n=3) were repeated three times in triplicates. *$p<0.05$.

Supplementary Fig. S3

A. MTS assay was used to measure cell viability in response to HNF1α overexpression in HPAC cells. **D.** Expression levels of pAKT, pmTOR, pERK, mTOR, pP70S6K, and β-actin were determined by Western Blot in overexpressed HNF1α HPAC cells. Data shown as mean +/- SEM. Experiments (n=3) were repeated three times in triplicates.

Supplementary Fig. S4

A & B. Apoptosis analysis of overexpressed HNF1α HPAC cells measured by flow cytometry. **C.** Western blot analysis of apoptotic markers in siHNF1α AsPC-1 cells and overexpressed HPAC cells. Data shown as mean +/- SEM. Experiments (n=3) were repeated three times in triplicates.

Supplementary Fig. S5

A & B. Wound-healing assay was performed in HNF1α overexpressed HPAC cells; migration was analyzed using Nikon Biostation Ct at 2h intervals for up to 96h at 4X magnification. **C & D.** Invasiveness of OVHNF1α HPAC cells were observed using a Matrigel invasion assay, captured using Nikon Eclipse TS 100 microscope. Data shown as mean +/- SEM. Experiments (n=3) were repeated three times in triplicates. *p<0.05.

Supplementary Fig. S6

A & B. Colony formation assay was performed in HNF1α overexpressed HPAC cells. **C.** EMT markers analyzed by Western blot in overexpression HNF1α in HPAC cells. Data shown as mean +/- SEM. Experiments (n=3) were repeated three times in triplicates. *p<0.05

Fig. 2A

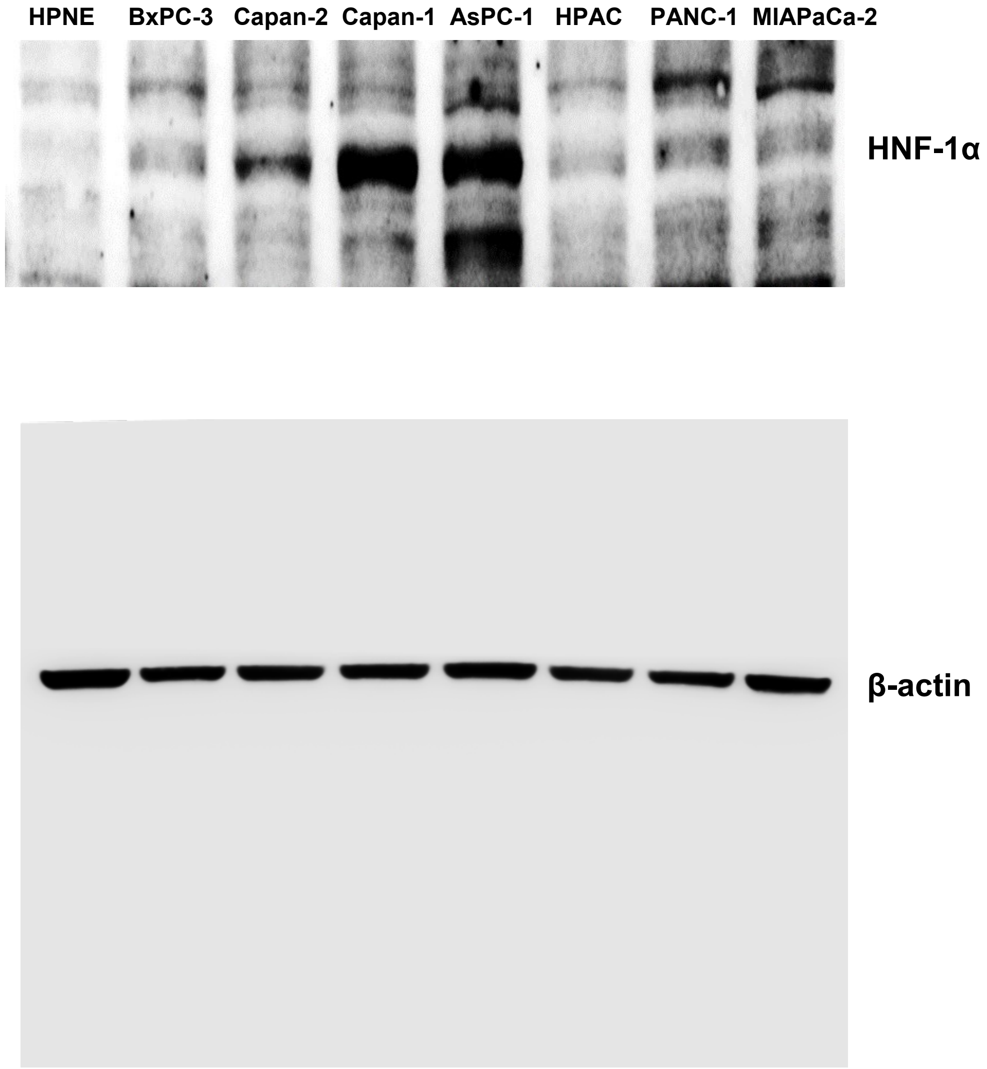
HPNE
BxPC-3
Capan-2
Capan-1
AsPC-1
HPAC
PANC-1
MIAPaCa-2
HNF-1α
β-actin

Fig. 2C

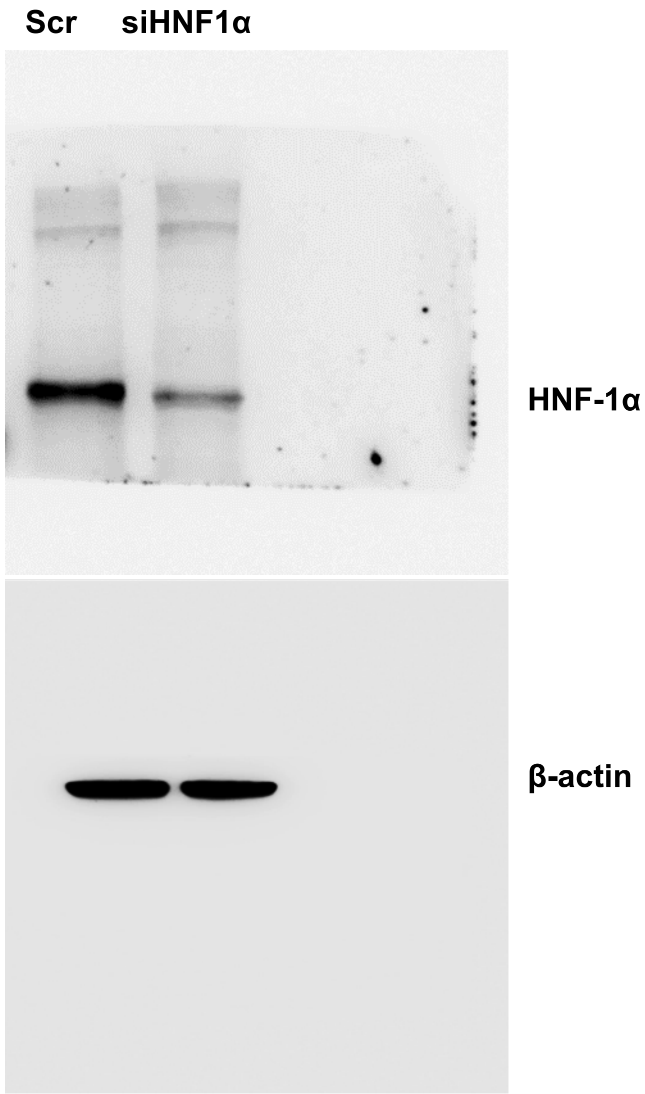
Scr
siHNF1α
HNF-1α
β-actin

Fig. 3A

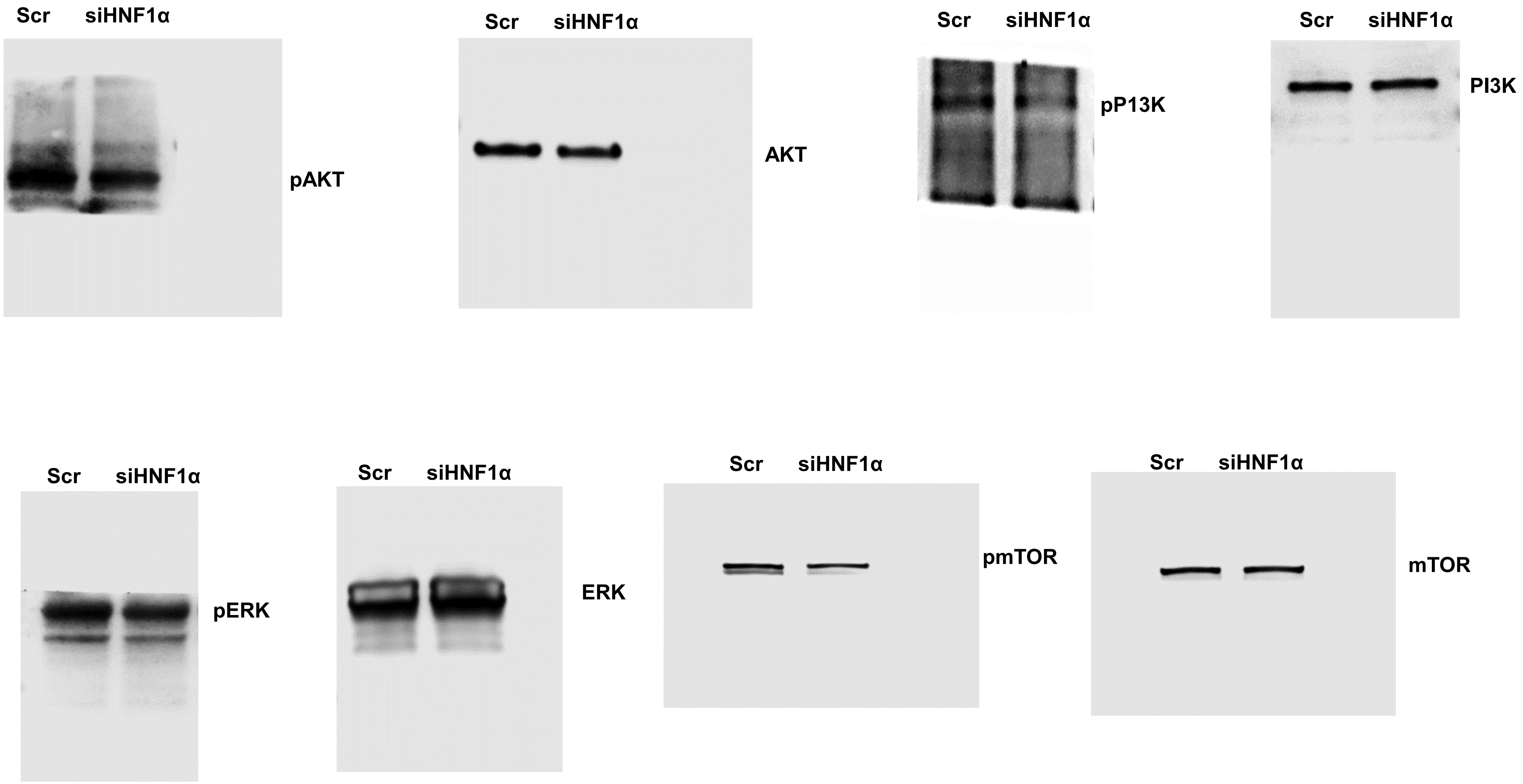
Scr
siHNF1α
pAKT
Scr
siHNF1α
AKT
Scr
siHNF1α
pP13K
Scr
siHNF1α
PI3K
Scr
siHNF1α
pERK
Scr
siHNF1α
ERK
Scr
siHNF1α
pmTOR
Scr
siHNF1α
mTOR

Fig. 3E

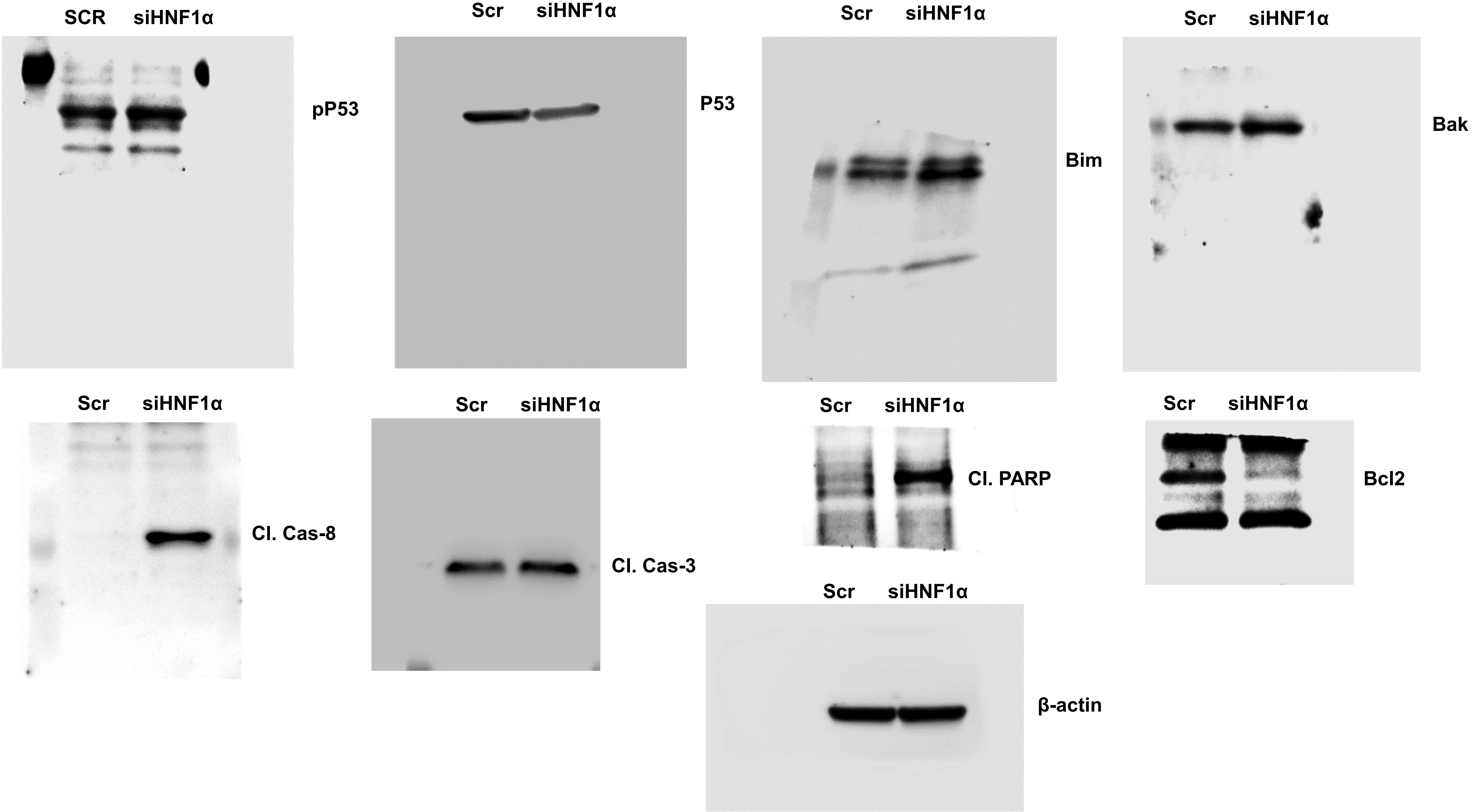
SCR
siHNF1α
pP53
Scr
siHNF1α
P53
Scr
siHNF1α
Bim
Scr
siHNF1α
Bak
Scr
siHNF1α
Cl. Cas-8
Scr
siHNF1α
Cl. Cas-3
Scr
siHNF1α
Cl. PARP
Scr
siHNF1α
Bcl2
Scr
siHNF1α
β-actin

Fig. 4G

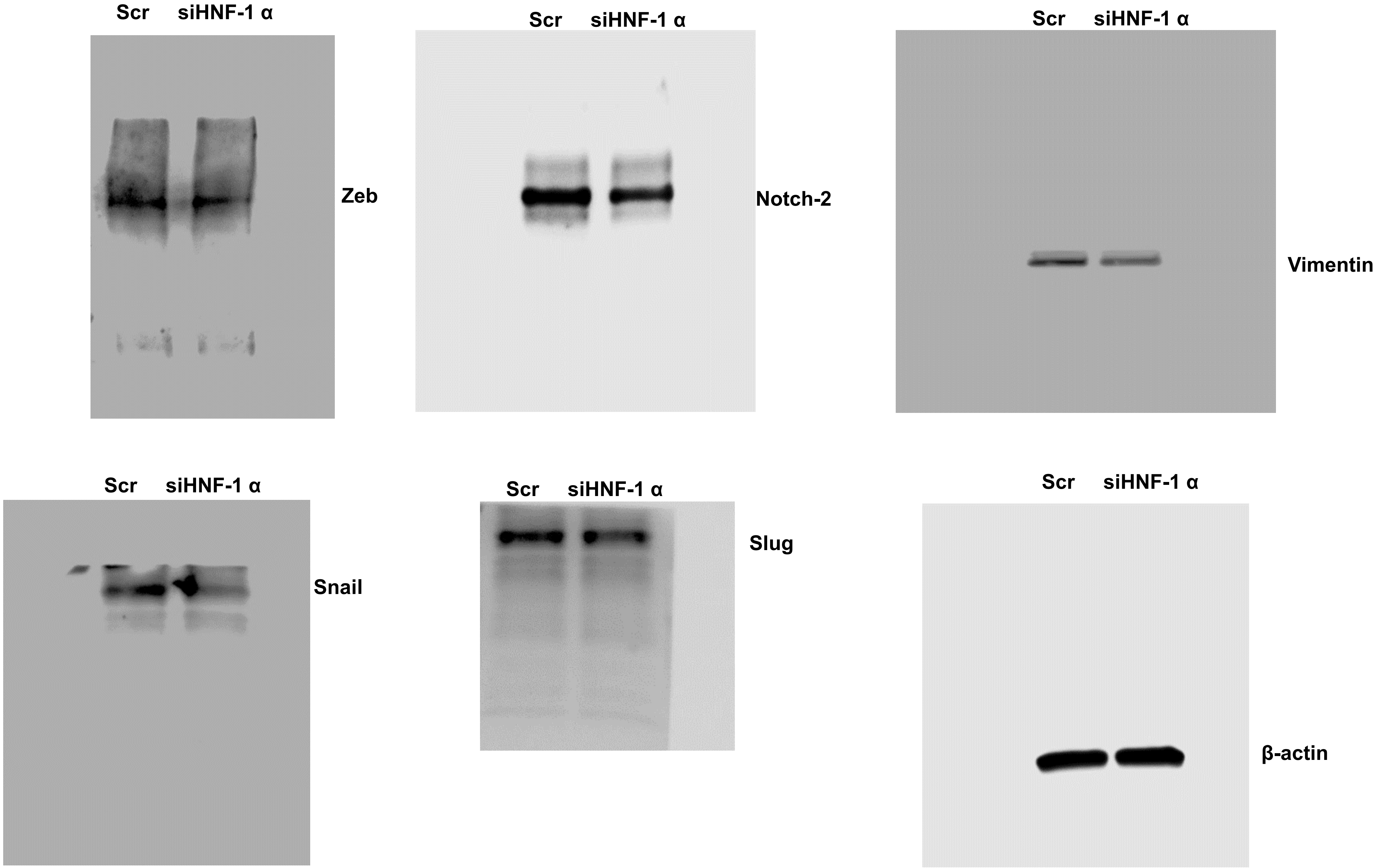

Scr
siHNF-1 α
Zeb
Scr
siHNF-1 α
Notch-2
Scr
siHNF-1 α
Vimentin
Scr
siHNF-1 α
Snail
Scr
siHNF-1 α
Slug
Scr
siHNF-1 α
β-actin

Fig. 5D

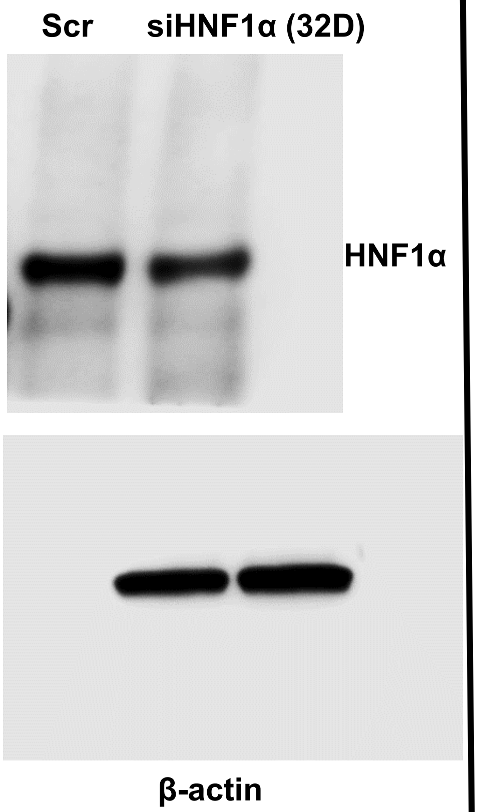
Scr
siHNF1α (32D)
HNF1α
β-actin

Fig. 5E

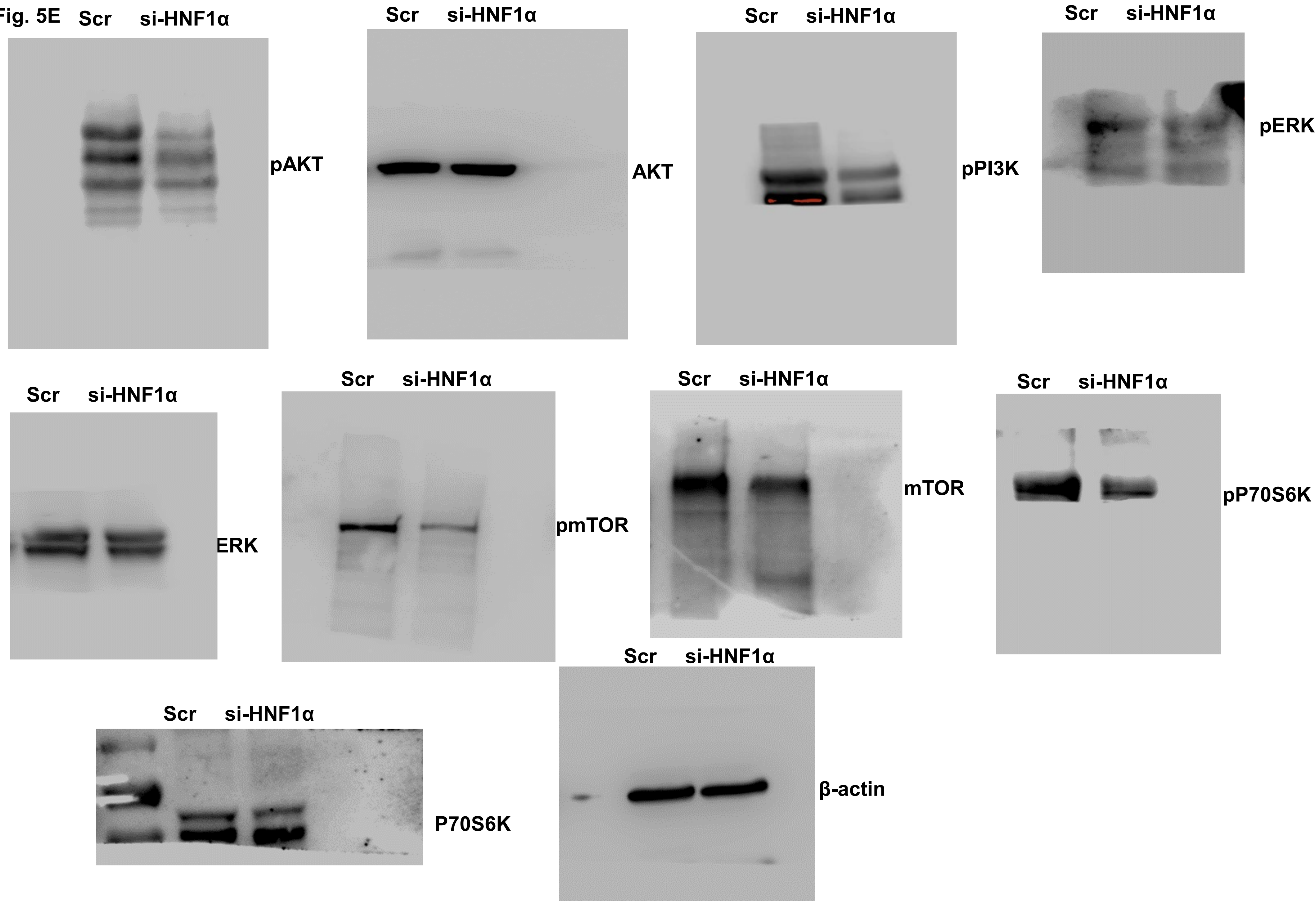
Scr
si-HNF1α
pAKT
AKT
pPI3K
pERK
ERK
pmTOR
mTOR
pP70S6K
P70S6K
β-actin

Fig. 5F

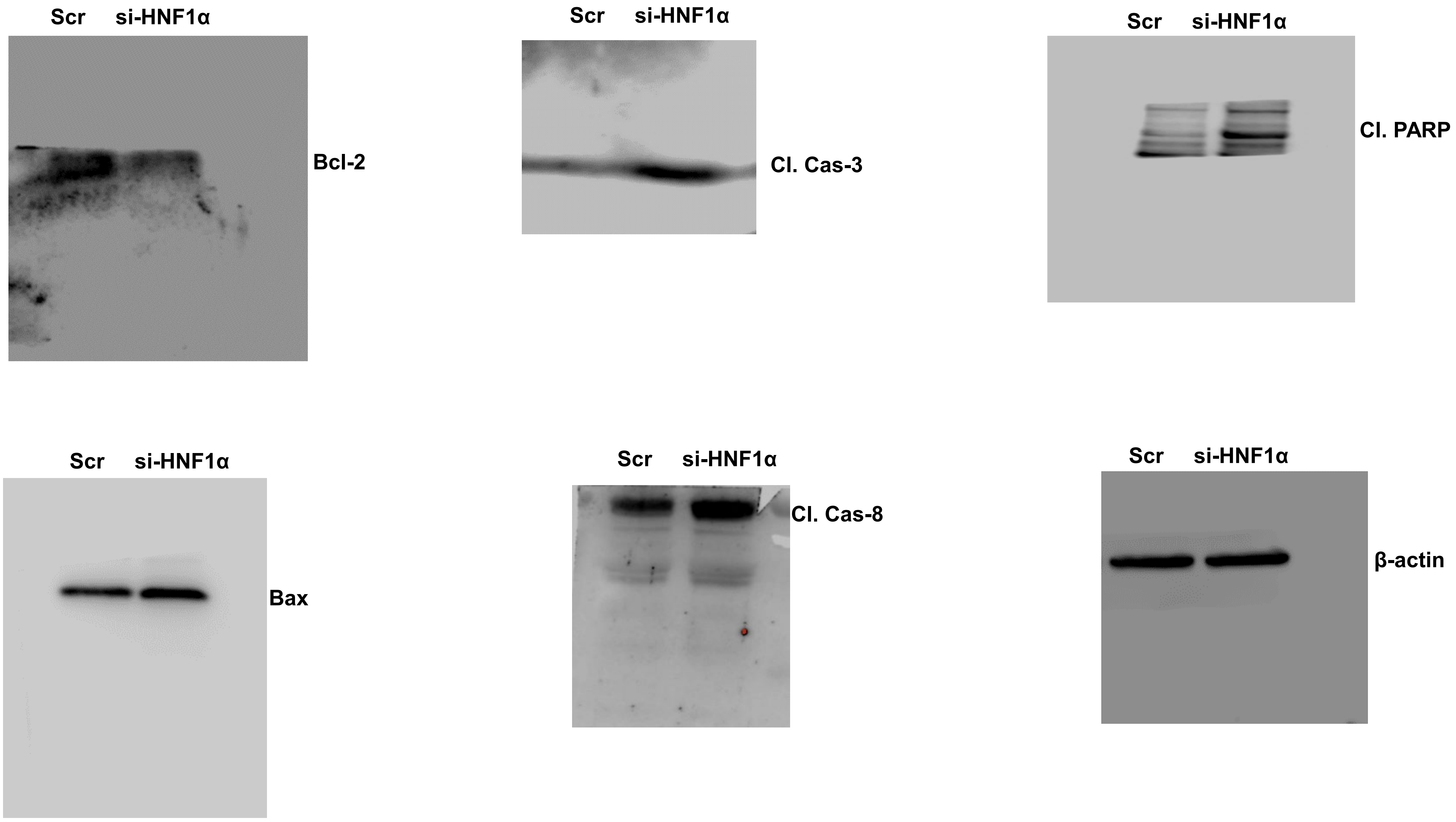
Scr
si-HNF1α
Bcl-2
Scr
si-HNF1α
Cl. Cas-3
Scr
si-HNF1α
Cl. PARP
Scr
si-HNF1α
Bax
Scr
si-HNF1α
Cl. Cas-8
Scr
si-HNF1α
β-actin

Fig. 5G

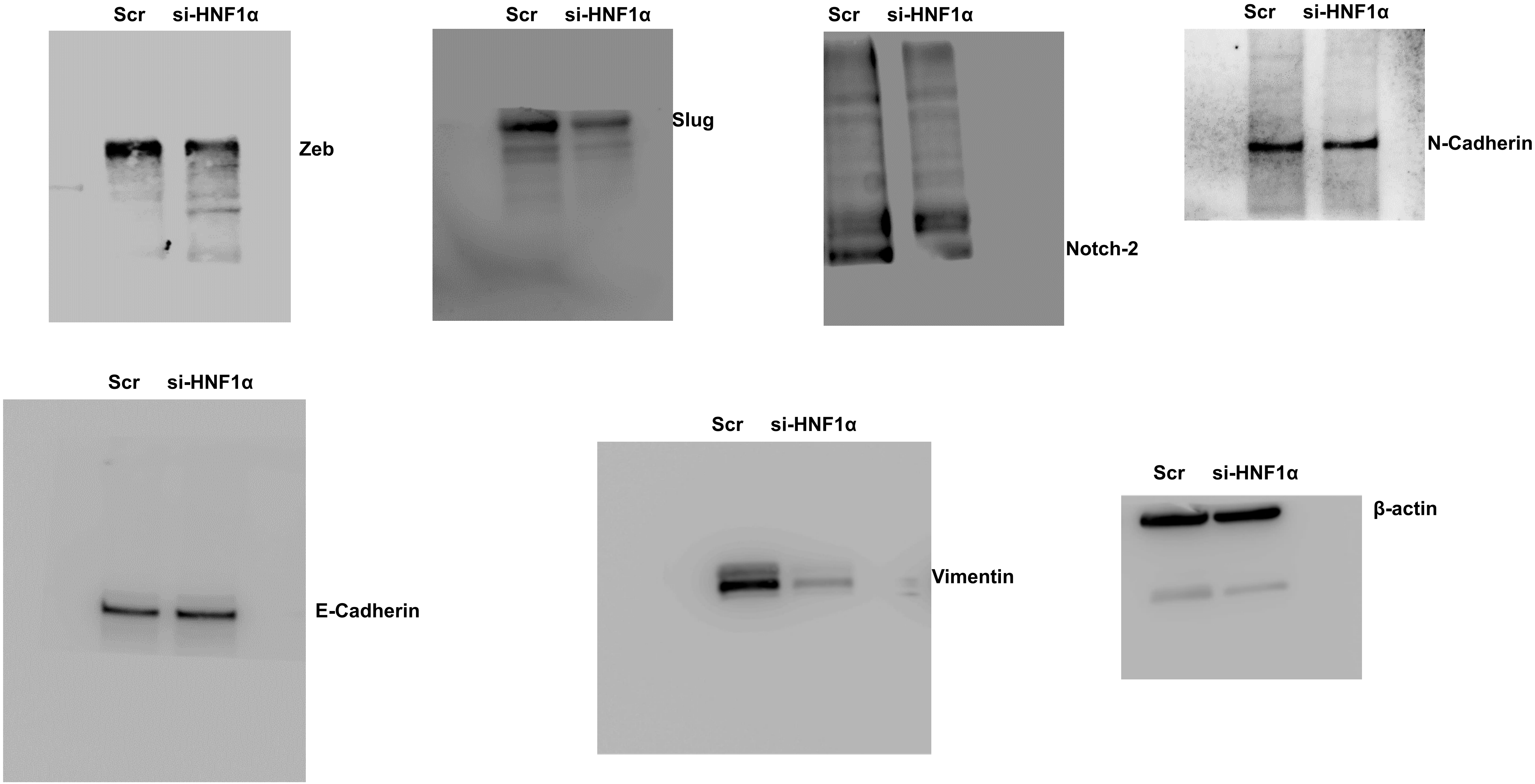
Scr
si-HNF1α
Zeb
Scr
si-HNF1α
Slug
Scr
si-HNF1α
Notch-2
Scr
si-HNF1α
N-Cadherin
Scr
si-HNF1α
E-Cadherin
Scr
si-HNF1α
Vimentin
Scr
si-HNF1α
β-actin

Supplementary Figure S1B

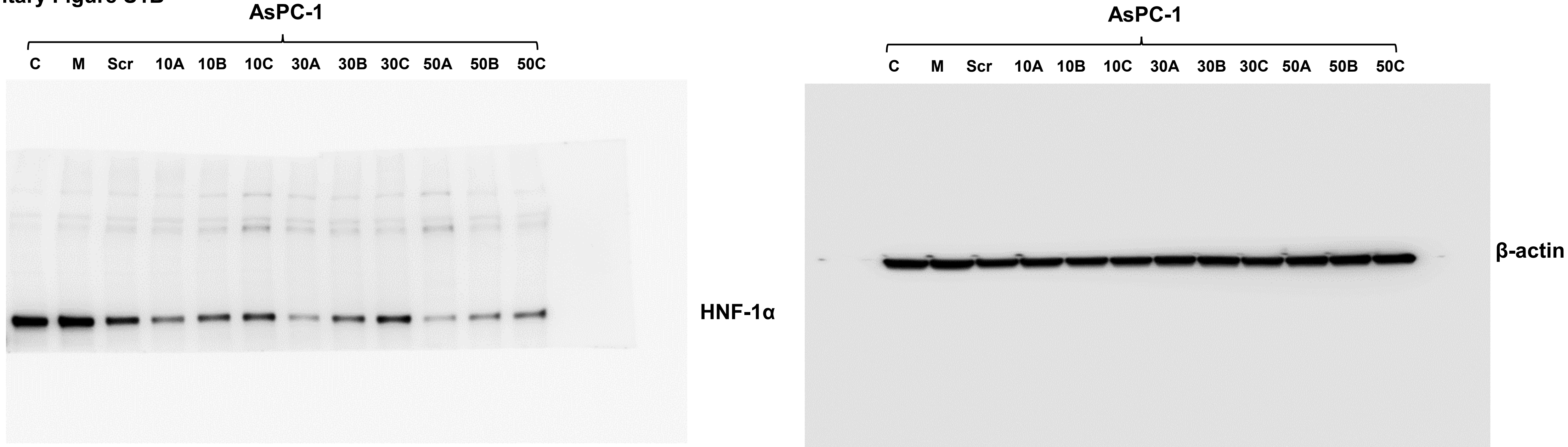

Supplementary Figure S2A

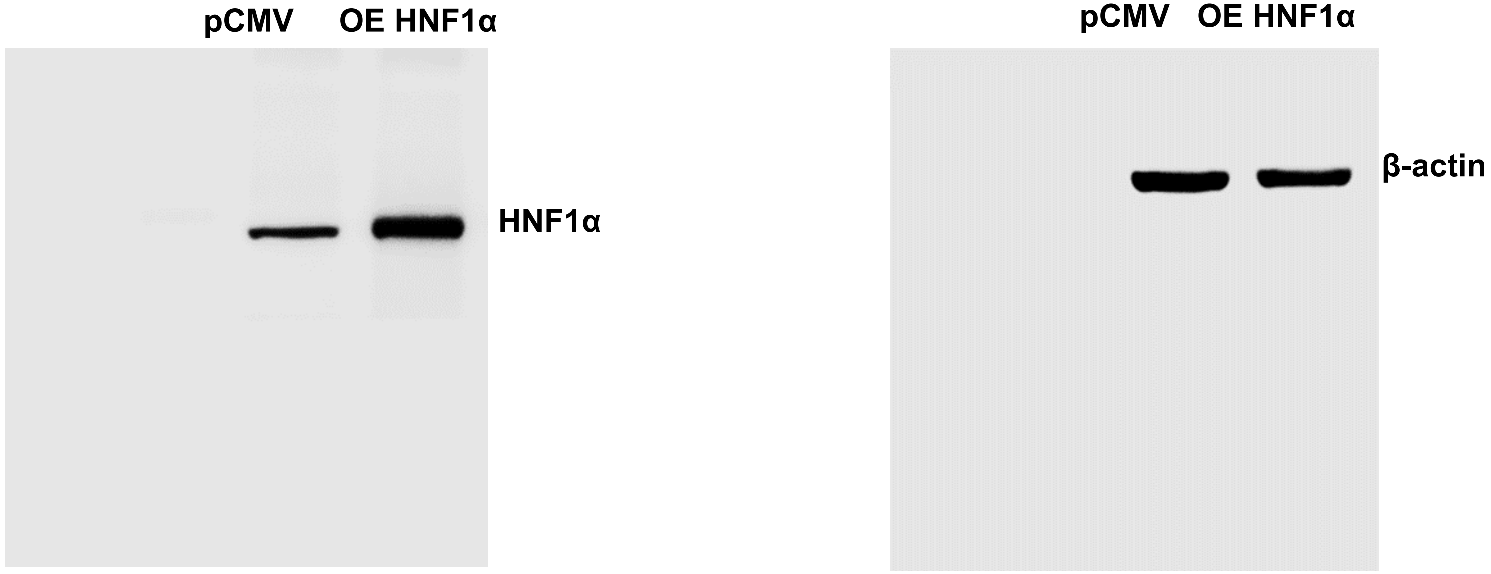

Supplementary Figure S3B

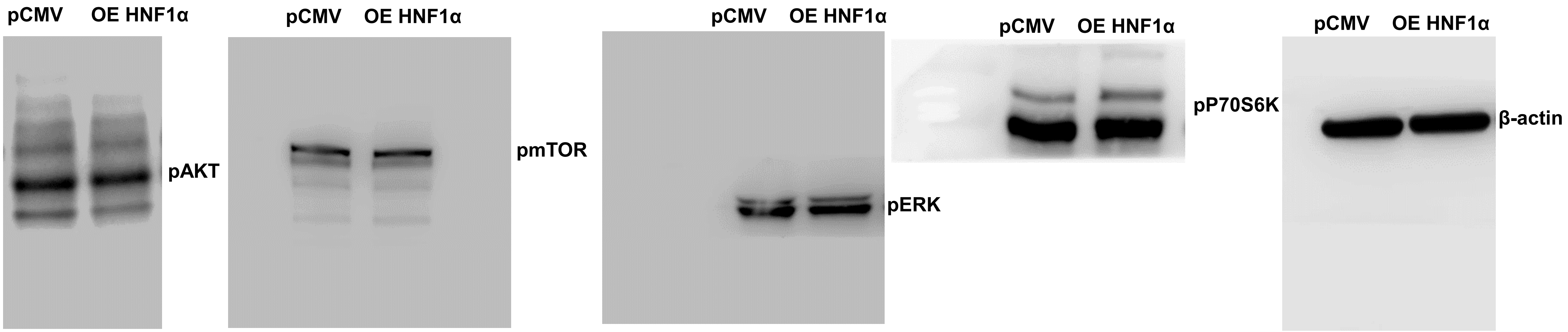

Supplementary Figure S4C

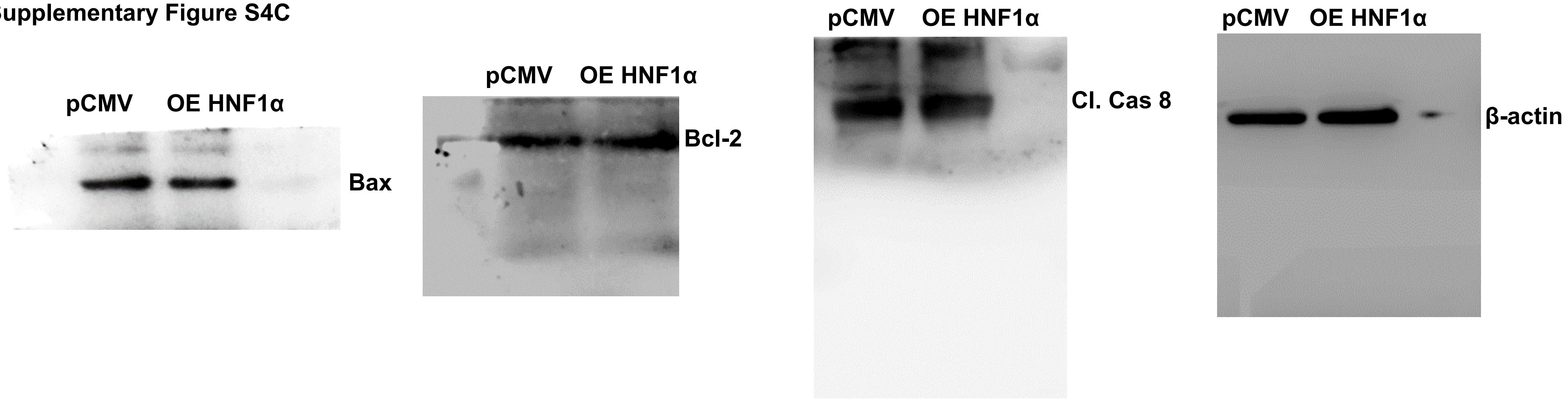

Supplementary Figure S6C

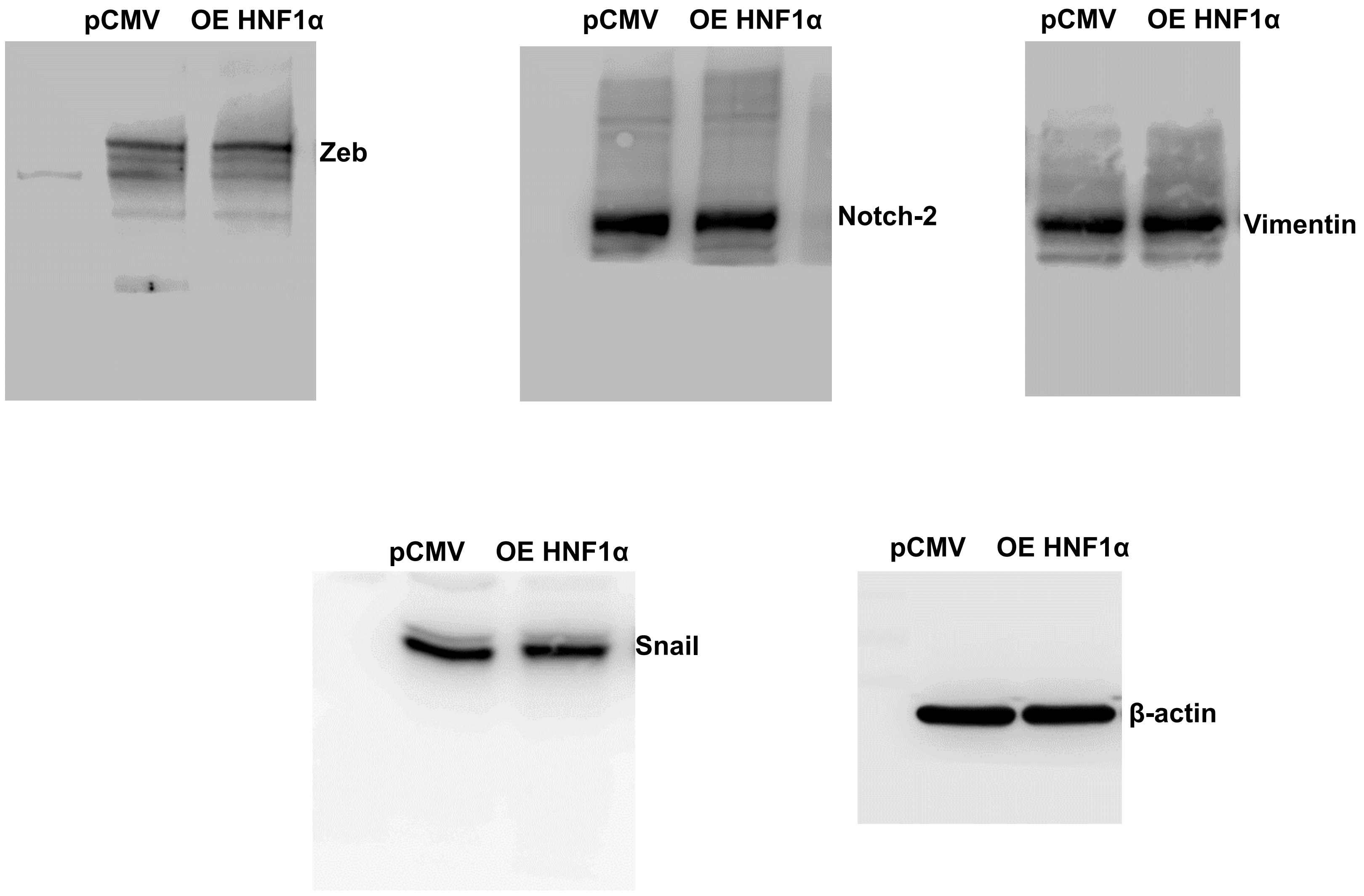

Supplement: Supplementary file 1 — Supplementary Information. [file 41598_2020_77287_MOESM1_ESM.pdf]
